# Protein Modification Characteristics of the Malaria Parasite *Plasmodium falciparum* and the Infected Erythrocytes

**DOI:** 10.1074/mcp.RA120.002375

**Published:** 2020-11-24

**Authors:** Jianhua Wang, Ning Jiang, Xiaoyu Sang, Na Yang, Ying Feng, Ran Chen, Xinyi Wang, Qijun Chen

**Affiliations:** 1 Key Laboratory of Livestock Infectious Diseases in Northeast China, Ministry of Education, Shenyang Agricultural University, Shengyang, China; 2 The Research Unit for Pathogenic Mechanisms of Zoonotic Parasites, Chinese Academy of Medical Sciences, Shenyang, China; 3 College of Food Science, Shenyang Agricultural Sciences, Shenyang, China; 4 College of Basic Sciences, Shenyang Agricultural University, Shenyang, China

**Keywords:** *Plasmodium falciparum*, protein posttranslational modification, molecular function, regulation, ACT1, actin-1, CR1, complement receptor 1, FDR, false discovery rate, G6PD, glucose-6-phosphate-dehydrogenase, GO, Gene Ontology, GPA, glycophorin-A, HSP70, heat shock protein 70, IDC, intraerythrocytic development cycle, IMAC, immobilized metal ion affinity chromatographic, iRBCs, infected red blood cells, KEGG, Kyoto Encyclopedia of Genes and Genomes, p.i., postinvasion, MESA, mature parasite–infected erythrocyte surface antigen, MSP 1, merozoite surface protein 1, NCE, normalized collisional energy, NSI, nano spray ion, PHIST, *Plasmodium* helical interspersed subtelomeric, PK, pyruvate kinase, PSM, propensity score matching, PTM, posttranslational modifications, pRBCs, *Plasmodium falciparum*–infected red blood cells, TMT, tandem mass tag, UPS, ubiquitin-associated proteasome system

## Abstract

Malaria elimination is still pending on the development of novel tools that rely on a deep understanding of parasite biology. Proteins of all living cells undergo myriad posttranslational modifications (PTMs) that are critical to multifarious life processes. An extensive proteome-wide dissection revealed a fine PTM map of most proteins in both *Plasmodium falciparum*, the causative agent of severe malaria, and the infected red blood cells. More than two-thirds of proteins of the parasite and its host cell underwent extensive and dynamic modification throughout the erythrocytic developmental stage. PTMs critically modulate the virulence factors involved in the host–parasite interaction and pathogenesis. Furthermore, *P. falciparum* stabilized the supporting proteins of erythrocyte origin by selective demodification. Collectively, our multiple omic analyses, apart from having furthered a deep understanding of the systems biology of *P. falciparum* and malaria pathogenesis, provide a valuable resource for mining new antimalarial targets.

## Highlights

• Protein posttranslational modifications of *Plasmodium falciparum* and erythrocytes were analyzed.
• Histones were regulated by acetylation; transcription and translation initiation were regulated by phosphorylation.
• Molecules in host-parasite interactions were extensively modified after translation.

## Graphical Abstract

**Figure:**
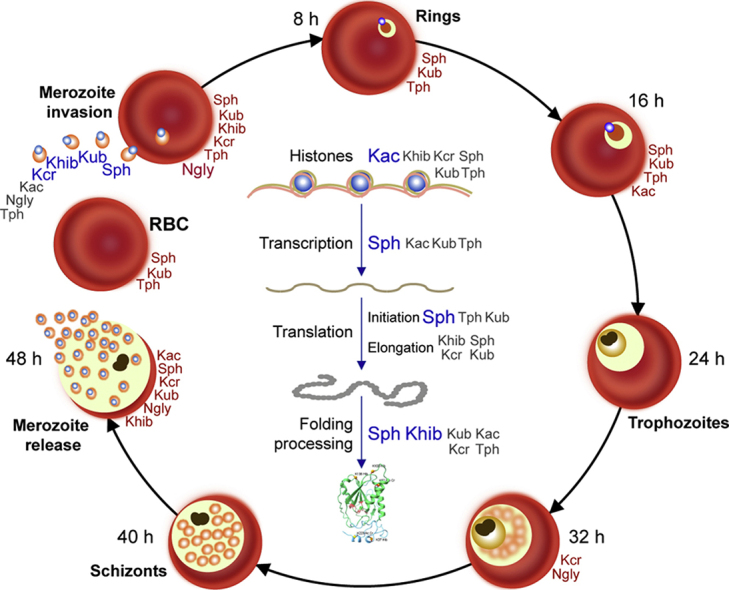

## In Brief

Tandem mass tag labeling, MS-based proteomics, and posttranslational modification (PTM)-omics were employed to identify samples of *Plasmodium falciparum* and its host erythrocytes at six time points, with healthy erythrocytes as controls. Dynamic modification levels of six PTM-omics (phosphorylation, acetylation, crotonylation, 2-hydroxyisobutyrylation, *N*-glycosylation, and ubiquitination) were comprehensively analyzed. Our research involved in the biology of malaria parasites from the overall PTM-omics level, laying an important foundation for pathogenic biology, cell biology, and drug mining of new antimalarial drugs.

Malaria remains one of the world's deadliest diseases, with an estimated 405,000 deaths each year; 67% of these are children under the age of 5 years. *Plasmodium falciparum* is the most prevalent malaria parasite in the World Health Organization's African Region, accounting for an estimated 99.7% of malaria cases in 2018 (1). The emergence of artemisinin-resistant strains poses a huge challenge for disease control because of the lack of successful vaccines and other effective antimalarials (2). *P. falciparum* undergoes a complex extraerythrocytic and intraerythrocytic development cycle that requires intricate and flexible regulatory mechanisms to maintain the parasite's resistance to environmental stress and facilitate reproduction. Sporozoites are inoculated into human circulation during the bite of an infected female *Anopheles* mosquito. The parasite infects hepatocytes and undergoes a liver stage that typically lasts for 1 week before the onset of the blood-stage development with serial cycles of asexual replication, and hence, human disease occurs (3). *P. falciparum* takes approximately 48 h to complete the intraerythrocytic development cycle (IDC) comprising three successive morphological stages (ring, trophozoite, and schizont stages) (4). A small proportion of parasites will develop into gametocytes that infect mosquitoes and continue the sexual development cycle (5). The interaction between *P. falciparum* and human erythrocytes is essential for cell invasion, intracellular development (6), and immune evasion, all of which are mediated by parasite-derived proteins.

Protein posttranslational modifications (PTMs) are essential processes following biogenesis that allow proteins to fulfill their diverse biological functions. PTMs occur in almost all cell types, and the regulatory elements are potential targets for drug mining (7, 8, 9). Previous studies on *P. falciparum* implicated the regulatory importance of PTMs in parasite development. Protein acetylation, phosphorylation, and ubiquitination regulate a wide range of general cellular activities such as DNA synthesis, signaling processes, protein activation, transcription, and metabolism, as well as key parasite processes such as invasion and cytoadherence (10, 11, 12, 13, 14, 15, 16, 17). Further, dynamic modification of histones maps the temporal patterns of 12 sites of H3 and H4 during the IDC of the parasite (18), implicating the critical roles of PTMs in regulation of parasite development. However, our current understanding of PTM on the regulatory mechanisms of parasite development and pathogenesis is still fragmentary rather than dynamic. In addition, 2-hydroxyisobutylation, crotonylation, and glycosylation have never been studied in *Plasmodium*, and information concerning the spontaneous modification of the proteins of infected red blood cells (iRBCs) is sparse (19).

Herein, we systematically analyzed six types of PTM-omics of both *P. falciparum* and iRBCs at six time points (8, 16, 24, 32, 40, and 48 h after invasion), using normal erythrocytes as controls. Our analyses provide a comprehensive and in-depth view of the dynamic PTM networks governing the development of the deadly malaria parasite throughout the IDC. This study provides a proteome and PTM-ome resource for *Plasmodium* parasites, and the data provide a foundation for the development of novel antimalarial drugs.

## Experimental Procedures

### Experimental Design and Statistical Rationale

Global proteome, phosphorylation, acetylation, crotonylation, 2-hydroxyisobutyrylation, *N*-glycosylation, and ubiquitination proteins of *P. falciparum* and pRBCs derived from highly synchronized cultures every 8 h during the IDC (8, 16, 24, 32, 40, 48 h, taking healthy erythrocytes as controls) were analyzed using tandem mass tag (TMT) labeling MS. Samples with three biological replicates were treated by protein extraction, pancreatin hydrolysis, TMT-10plex labeling, HPLC, and enrichment followed by LC-MS analysis. The quality of protein samples was detected using SDS-PAGE, and three biological replicates were analyzed in each experiment to validate the biological reliability of measurements. Log2 geometric means of normalized expression values of the three replicates were used to indicate the abundance of a protein or a modification site at a specific time point. TMT ratios of proteins or PTM sites were calculated by the median of all of the propensity score matching (PSM) ratios, excluding the PSMs lacking TMT labels.

### Cultivation of *P. falciparum* 3D7 Strain Parasites and Sample Preparation

Highly synchronized cells of *P. falciparum* strain 3D7 were cultured at 5% parasitemia. iRBCs were obtained by a Percoll-sorbitol purification method at 8, 16, 24, 32, 40, and 48 h during the intracellular cycle. This method greatly improved the purity of *P. falciparum*-infected RBCs, but the parasitemia did not reach 100% and was not the same at each time point. To solve this problem, we used the median normalization method for standardization, so that the differences in parasitemia did not affect the accuracy of the results. Samples in lysis buffer (8 M urea, 1% protease inhibitor cocktail) were sonicated three times on ice. The samples were centrifugated at 12,000*g*, 4 °C for 10 min. The supernatants were collected, and the protein concentration of each sample was determined using a bicinchoninic acid kit according to the manufacturer's instructions. The quality of protein samples was detected using SDS-PAGE.

### Western Blotting Analysis

Western blotting analysis was performed using standard procedures for whole-cell extracts. Antibodies used were antiacetyllysine mouse mAb (PTM-101) for Kac, anticrotonyllysine mouse mAb (PTM-502) for Kcr, antiphosphotyrosine mouse mAb (PTM-701) for Yph, anti-2-hydroxyisobutyryllysine rabbit pAb (PTM-801) for Khib, antiubiquitin rabbit pAb (PTM-1106) for Kub, and HRP-conjugated goat anti-Mouse IgG (H + L) secondary antibody (1:10,000, Thermo Fisher Scientific).

### Trypsin Digestion

For trypsin digestion, the protein solution was reduced with 5 mM dithiothreitol for 30 min at 56 °C and alkylated with 11 mM iodoacetamide for 15 min at room temperature. The urea concentration of the sample was diluted to less than 2M by adding 100 mM triethylamine bicarbonate. Finally, trypsin was added at a 1:50 trypsin-to-protein mass ratio for the first step overnight digestion, and a second 4-h digestion with additional trypsin was performed with a 1:100 trypsin-to-protein mass ratio.

### HPLC Fractionation

The trypsin-digested peptides were fractionated by high pH reverse-phase HPLC using an Agilent 300 Extend C18 column (5-μm particles, 4.6 mm ID, 250 mm length). Briefly, peptides were first separated with a gradient of 8%–32% acetonitrile (pH 9.0) over 60 min into 60 fractions. Then, the peptides were combined into 18 fractions and dried by vacuum centrifuging.

### Pan Antibody-Based PTM Enrichment (for Acetylation, Crotonylation, 2-Hydroxyisobutyrylation, and Ubiquitination)

The trypsin-digested peptides dissolved in NETN buffer (100 mM NaCl, 1 mM EDTA, 50 mM Tris-HCl, 0.5% NP-40, pH 8.0) were incubated with prewashed antibody-conjugated beads (PTM-104 for acetylation, PTM-503 for crotonylation, PTM-804 for 2-hydroxyisobutyrylation, and PTM-1104 for ubiquitination, PTM Bio) at 4 °C overnight with gentle shaking. Then, the beads were washed four times with NETN buffer and twice with H_2_O. The bound peptides were eluted from the beads with 0.1% trifluoroacetic acid. Finally, the eluted fractions were combined and vacuum-dried. For LC-MS/MS analysis, the resulting peptides were desalted with C18 ZipTips (Millipore) according to the manufacturer's instructions.

### Biomaterial–Based PTM Enrichment (for Phosphorylation)

Peptide mixtures were first incubated with an immobilized metal ion affinity chromatographic (IMAC) microsphere suspension with vibration. The IMAC microspheres with enriched phosphopeptides were collected by centrifugation, and the supernatant was removed. To remove nonspecifically adsorbed peptides, the IMAC microspheres were washed with 50% ACN/6% TFA and 30% ACN/0.1% TFA sequentially. To elute the enriched phosphopeptides from the IMAC microspheres, elution buffer containing 10% NH_4_OH was added. The supernatant containing phosphopeptides was collected and lyophilized for LC-MS/MS analysis.

### Hydrophilic Interaction Chromatography-Based PTM Enrichment (for *N*-Glycosylation)

The peptides were dissolved in 40 μl of enrichment buffer (80% acetonitrile/1% trifluoroacetic acid), and the supernatant was transferred to a hydrophilic interaction chromatography microcolumn. Enrichment was completed by centrifugation at 4000*g* for approximately 15 min. The hydrophilic microcolumn was washed three times with enrichment buffer. The glycopeptide was then eluted with 10% acetonitrile, and the eluate was collected and vacuum dried. After drying, the eluate was resuspended in 50 μl of 50 mM ammonium bicarbonate buffer dissolved in hydrogen peroxide solution. Following this, 2 μl of PNGase F glycosidase was added, and the eluate was cleaved overnight at 37 °C. Finally, the salt was removed according to the C18 ZipTips instructions.

### LC-MS/MS Analysis for Proteome

The tryptic peptides were dissolved in 0.1% formic acid (solvent A) and directly loaded onto a homemade reversed-phase analytical column. The gradient of solvent B (0.1% formic acid in 90% acetonitrile) comprised an increase from 9% to 24% over 38 min, 24% to 35% in 14 min, and climbing to 80% in 3 min, then holding at 80% for the last 3 min, all at a constant flow rate of 700 nl/min on an EASY-nLC 1000 ultra-high-performance chromatography (UPLC) system. The peptides were subjected to nano spray ion (NSI) source followed by tandem mass spectrometry (MS/MS) in a Q ExactiveTM Plus (Thermo) coupled online to the UPLC. The electrospray voltage applied was 2.0 kV. The m/z scan range was 350 to 1800 for full scans, and intact peptides were detected in the Orbitrap at a resolution of 70,000. Peptides were then selected for MS/MS using an NCE setting of 30, and the fragments were detected in the Orbitrap at a resolution of 17,500. The signal threshold was set to 10,000 ions/s, and the maximum injection time was set to 200 ms. The dynamic exclusion time of the MS/MS scan was set to 30 s to avoid repeated scans of the parent ions. There are corresponding Excel tables for each peptide sequence of three biological replicates (supplemental Datasets S1–S3).

### LC-MS/MS Analysis for Phosphorylation

The peptides were subjected to NSI source followed by MS/MS in a Q ExactiveTM Plus (Thermo) coupled online to the UPLC (supplemental Datasets S4–S6). The electrospray voltage applied was 2.0 kV. The m/z scan range was 350 to 1600 for the full scan, and intact peptides were detected in the Orbitrap at a resolution of 60,000. Peptides were then selected for MS/MS using a normalized collisional energy (NCE) setting of 28, and the fragments were detected in the Orbitrap at a resolution of 15,000. A data-dependent procedure that alternated between one MS scan followed by 20 MS/MS scans with 15.0s dynamic exclusion was employed. The fixed first mass was set as 100 m/z. Automatic gain control was set at 1E5. The signal threshold was set to 33,000 ions/s, and the maximum injection time was set to 60 ms. The dynamic exclusion time of the MS/MS scan was set to 15 s to avoid repeated scans of the parent ions.

### LC-MS/MS Analysis for Acetylation, Crotonylation, 2-Hydroxyisobutyrylation, and Ubiquitination

The peptides were subjected to NSI source followed by MS/MS in a Q ExactiveTM Plus (Thermo) coupled online to the UPLC (supplemental Datasets S7–S18). The electrospray voltage applied was 2.0 kV. The m/z scan range was 350 to 1600 for the full scan, and intact peptides were detected in the Orbitrap at a resolution of 60,000. Peptides were then selected for MS/MS using an NCE setting of 28, and the fragments were detected in the Orbitrap at a resolution of 30,000. A data-dependent procedure that alternated between one MS scan followed by 20 MS/MS scans with 15.0 s dynamic exclusion was employed. Automatic gain control was set at 1E5. The fixed first mass was set as 100 m/z. The signal threshold was set to 20,000 ions/s, and the maximum injection time was set to 200 ms. The dynamic exclusion time of the MS/MS scan was set to 30 s to avoid repeated scans of the parent ions.

### LC-MS/MS Analysis for *N*-Glycosylation

The tryptic peptides were dissolved in 0.1% formic acid (solvent A) and directly loaded onto a homemade reversed-phase analytical column (15 cm length, 75 μm i.d.). The gradient of solvent B (0.1% formic acid in 90% acetonitrile) comprised an increase from 8% to 23% over 38 min, 23% to 35% in 14 min, climbing to 80% in 3 min, then holding at 80% for the last 3 min, all at a constant flow rate of 450 nl/min on an EASY-nLC 1000 UPLC system. Corresponding Excel tables for each peptide sequences of three biological replicates were provided (supplemental Datasets S19–21).

### Database Search

The resulting MS/MS data were processed using the MaxQuant search engine (v.1.5.2.8). Tandem mass spectra were searched against SwissProt *Homo sapiens* (March 2018, containing 20,317 sequences) and UniProt *P. falciparum* (isolate 3D7) (www.PlamoDB.org, March 2018, containing 5369 sequences), along with reverse decoys and a standard contaminants database from MaxQuant. The reverse decoy database was used to calculate the false positive rate (FDR) caused by random matching. The common contamination databases were used to eliminate the effects of contaminating proteins in the identification data. Trypsin/P was specified as a cleavage enzyme, allowing up to four missing cleavages for acylation and ubiquitination and two missing cleavages for phosphorylation and glycosylation. The minimum length of the peptide was set to seven amino acid residues, and the maximum number of peptide modifications was set to five. The mass tolerance for precursor ions was set as 20 ppm in the First search and 5 ppm in the Main search, and the mass tolerance for fragment ions was set as 0.02 Da. Carbamidomethyl on cysteine was specified as a fixed modification. Oxidation of methionine and acetylation of the N terminals of proteins were specified as variable modifications. In addition, variable modifications were specified for phosphorylation (of serine, threonine, and tyrosine), acetylation, crotonylation, 2-hydroxyisobutyrylation and ubiquitination (with TMT tag on lysine), deamidation 18O (N), and deamidation (NQ). The FDR for protein identification and PSM identification was adjusted to less than 1%, and the quantitative method was set to TMT-10plex. Unique and razor peptides were selected for quantification, and the minimum ratio count was set to 2. The minimum score for modifying peptides was kept at 40, and the localization probability was greater than 0.75.

### Quality Control of Mass Spectrometry

Primary mass errors of most spectra were less than 10 ppm, which was consistent with the high-precision characteristics of orbitrap mass spectrometry. This indicated that the quality accuracy of the mass spectrometer was normal and that there was no excessive mass deviation to affect the qualitative or quantitative analyses of proteins. The match score between the spectrum and the peptide was negatively correlated with the distribution of the mass deviation. The higher the score, the smaller the quality deviation. This score characterized the credibility of peptide identification.

### Quality Control of Peptide Lengths

The sizes of most peptides ranged from 7 to 20 amino acids, consistent with the general rule of methods based on trypsin enzymatic and higher-energy collisional dissociation fragmentation. Peptides with less than five amino acids produced too few fragment ions and therefore did not produce efficient sequence identification. Peptides with more than 20 amino acids were not suitable for fragmentation of higher-energy collisional dissociation because of the high mass and charge number. In other words, the distribution of peptide lengths identified by MS met quality control requirements.

For biologically replicated and technically replicated samples, we examined whether the quantitative results of biological replicates or technical replicates were statistically consistent. Heatmaps were generated using Pearson's correlation coefficients between all samples. This coefficient is a measure of the degree of linear correlation between the two sets of data: a Pearson coefficient close to −1 indicates a negative correlation, and a value close to 1 represents a positive correlation, while values near 0 indicate no correlation. In this experiment, Pearson's correlation coefficients were calculated for each sample of each modification. The results showed that the repeatability of the three biological replicates was good. In short, the accuracy of the test instrument was normal; the peptide quality control was suitable; the biological repeatability was good, and the data could be used for subsequent analysis.

### Quantification of Proteins or Modification Sites

Relative abundance of proteins or modification sites was determined by the ion intensity ratio of the TMT reporter from each PSM in MaxQuant. TMT ratios of proteins or PTM sites were calculated by the median of all of the PSM ratios, excluding the PSMs lacking TMT labels.

### Generation of Circos Plots

The Circos plots were generated using Circos (http://www.circos.ca) software. The panels were made in the program R using the custom package imsbInfer, currently on Github (https://github.com/wolski/imsbInfer). The quantified modified proteins were first sorted according to the number of modification types and then by the number of modification sites. The Log2 value of the geometric mean of normalized expression values (from the signal intensity of MS/MS) of the three repetitions was used to indicate the abundance of a protein or a modification site at a specific time point. The median normalization method was used to exclude the effects of ratio changes of parasites and RBCs in the mixed pRBCs sample over time on the abundance of proteins or modification sites. Changes in the Log2 geometric mean of normalized expression values for each protein or site were considered to be consistent with their actual abundance trends. The larger the value, the higher the abundance. The circle diagrams were generated by the circos of the histogram set of each protein at a specific time point in the proteome and the gradation diagram set of each modification site at a specific time point in PTM-omics. According to the initial order, the accumulated value of the amino acid length is displayed on the outer circle, so that the corresponding protein and its modification can be located, and thus, the outermost circle represents “cumulative protein length of identified proteins.”

### Significantly Changed Proteins or Modification Sites

The average ratio of proteins or modification sites at each time point during the IDC was used to describe the abundance, including the peak and the valley. The FDR was calculated based on an ANOVA proposed by Benjamini and Hochberg. An FDR of less than 1% was considered to be a significant difference.

### Dynamic Cluster Analysis

To perform cluster analyses of the modification sites with significantly changed abundance during the IDC, we used a clustering method based on the Mfuzz package, version 2.36.0 (https://www.bioconductor.org/packages/release/bioc/html/Mfuzz.html). This method allows multiassignment of quantified proteins or modification sites in different clusters. The number of clusters was set to six, and the fuzzification parameter *m* was set to 2. To identify the main functions of proteins in each cluster, we performed GO enrichment and pathway enrichment followed by adjustment of *P* values using R's p. adjust function.

### Subcellular Localization Prediction

The subcellular localization of proteins was predicted based on the amino acid sequence of the proteins modified by PTMs through the online tool WOLF PSORT (wolfpsort.org).

### Dynamic Analysis of PTM Abundance in Subcellular Localization

To explore the relationship between the PTM abundance and subcellular localization during the IDC, the enrichment score of subcellular localization was developed from the scoring tactics of dynamic changes of PTM abundance. Single-sample gene set enrichment analysis was used to calculate normalized enrichment scores of relative abundance of PTMs at each time point during the IDC.

### Functional Annotation and Enrichment

GO annotation of proteins was derived from the UniProt-GOA database (http://www.ebi.ac.uk/GOA/). The protein accession was first converted to the GO ID, and then the protein function was annotated with Uniprot-GOA. If the proteins were not annotated by the Uniprot-GOA database, the annotation was produced by the InterProScan software based on the sequence alignment of proteins. The KEGG database was used to annotate the pathways of proteins. The protein description from the KEGG pathway was annotated by the online service tools KAAS (KEGG automatic annotation server) and KEGG mapper in the KEGG database. The functional enrichment was analyzed using Fisher's exact test.

### Protein–Protein Interaction Network

The interaction among protein sets was searched using the STRING database, version 11.0 (https://string-db.org/), based on the protein accession or amino acid sequences of proteins. To ensure the credibility of the interaction, only trial-verified relationships were selected, so “Text mining” was removed in the settings. In STRING, a metric called “confidence score” is used to define interaction confidence. We required all relationships to have a high confidence score (>0.9 for nucleic acid-related proteins, >0.8 for disease pathway-related proteins). The interaction network was constructed based on the modification sites through Cytoscape.

### Construction of the 3D Structure

The 3D structures of the target proteins were downloaded from the Protein Data Bank (PDB; http://pdb101.rcsb.org/) database based on the amino acid sequences. The omnidirectional videos were generated by ChimeraX software (https://www.cgl.ucsf.edu/chimera/), and the modification sites were marked.

## Results

### The Overall PTM-omics of *P. falciparum* and Infected Erythrocytes

**Fig. 1:**
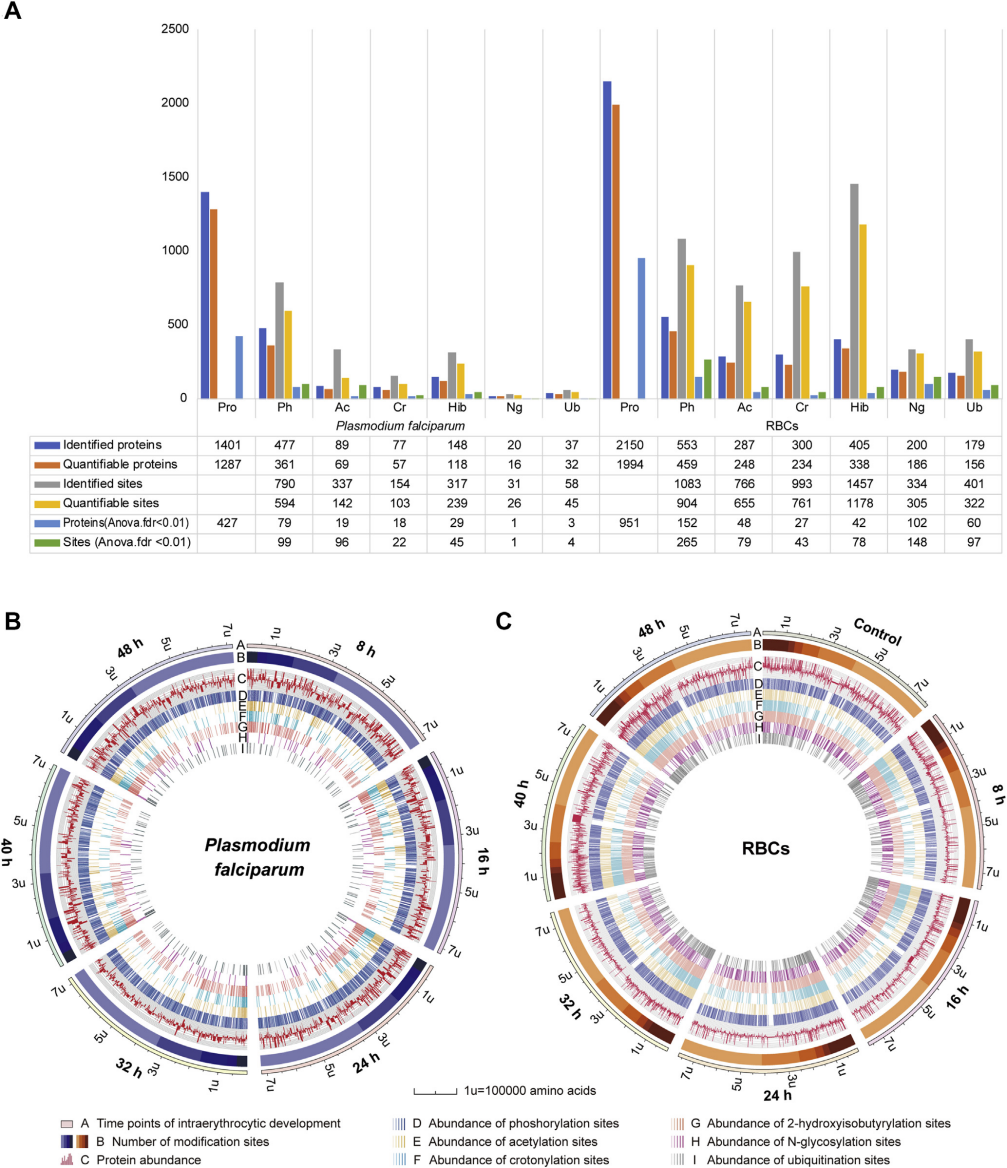
**Integration of proteome and six PTM-omics of *P. falciparum* and infected RBCs**. *A*, histograms of proteomes and six PTM-omics of *P. falciparum* and pRBCs. The number of identified proteins and modification sites with significant variation (FDR < 0.01) are included. *B*, circos plot showing the abundance configurations of proteomes and six modification omics at each time point for *P. falciparum* proteins. The levels of each modification site at six time points are represented by a gradient color based on the Log2 geometric mean of the normalized expression values of the three repetitions. The larger the Log2 geometric mean, the darker the color. 1U = 100,000 amino acid. *C*, circos plot showing the abundance configurations of proteome and six modification omics at each time point of the RBC proteins. Ac, acetylation; Cr, crotonylation; FDR, false discovery rate; Hib, 2-hydroxyisobutyrylation; Ng, *N*-glycosylation; Ph, phosphorylation; Ub, ubiquitination.

*P. falciparum*- and RBC-derived proteins were purified from highly synchronized cultures every 8 h postinvasion (p.i.), and the proteomic dynamics and turnover of six PTMs of each protein were thoroughly analyzed (supplemental Figs. S1 and S2; supplemental Dataset S22). For *P. falciparum*, we identified 1401 proteins and 1518 modification sites that were matched to 848 proteins in the six PTM-omics (phosphorylation, acetylation, crotonylation, 2-hydroxyisobutyrylation, *N*-glycosylation, and ubiquitination) in varying degrees across the erythrocytic stage (Fig. 1, *A–B*; supplemental Datasets S23 and S24). For the host RBCs, 5034 modification sites matched to 1924 proteins were identified (Fig. 1*C*; supplemental Datasets S23 and S24). Phosphorylation was the most predominant modification type in the *P. falciparum* proteome, whereas in the RBC proteins, apart from phosphorylation, acetylation, crotonylation, and 2-hydroxyisobutyrylation were prominent (Fig. 1, *A*–*C*). The variation in modification abundance was considered to be significant at the 1% false discovery rate (FDR) level.

Functional characterization based on Gene Ontology (GO) terms (*p* < 0.05 for *P. falciparum* and *p* < 0.00001 for RBCs) and the Kyoto Encyclopedia of Genes and Genomes (KEGG) pathways (*p* < 0.05 for *P. falciparum* and *p* < 0.01 for RBCs) (supplemental Fig. S3; supplemental Dataset S25) illustrated that the acetylated proteins in *P. falciparum* were predominantly located in the nucleus and ribosome, with functions such as DNA binding, protein heterodimerization activity, and organelle organization (supplemental Fig. S3*A*), in line with earlier findings in both *P. falciparum* and other organisms (8, 20, 21, 22). Lysine crotonylation and 2-hydroxyisobutyrylation occurred on proteins mainly located in the cytosol of both parasites and RBCs, mediating diverse metabolic pathways (supplemental Fig. S3, *A–B*), similar to our previous findings in *Toxoplasma gondii* (23). For the RBC proteins, acylation including acetylation, crotonylation, and 2-hydroxyisobutyrylation mainly occurred in proteins associated with sugar metabolism, such as in glycolysis/gluconeogenesis and pentose phosphate pathways, and oxidative stress, proteasome, *de novo* protein folding, hypoxia-inducible factor-1 signaling pathway, and cadherin binding. Proteins modified by 2-hydroxyisobutyrylation also participated in spectrin binding and disulfide oxidoreductase activity (supplemental Fig. S3*B*). In *P. falciparum*, phosphorylation mainly occurred on proteins involved in RNA transport, spliceosomes, mismatch repair, ribosomes, protein processing in the endoplasmic reticulum, the phosphatidylinositol signaling system, metabolic regulation, and response to drugs (supplemental Fig. S3*A*). In the RBC host cells, phosphorylated proteins were mainly involved in kinase activity, glycolysis/gluconeogenesis, pentose phosphate pathways, the citrate cycle, cadherin, actin-binding, and other processes (supplemental Fig. S3*B*). Ubiquitination is mostly known as a signal for proteasomal degradation, primarily participating in metabolic process and proteolysis (24), immune suppression, and host cell reprogramming (13). We found that in *P. falciparum*, proteins associated with RNA transport, translation, substance metabolism, cellular biosynthesis, and response to oxidative stress (supplemental Fig. S3*A*) were ubiquitinated, and in RBCs, ubiquitination mainly occurred in proteins associated with catabolic and antigenic processing, but ubiquitination of the erythrocytic structural proteins was significantly less than that in the normal RBCs (supplemental Fig. S3*B*). Proteins with *N*-glycosylation were mainly involved in cellular localization and protein binding in *P. falciparum* (supplemental Fig. S3*A*), but those events were much less frequent compared with other modifications. *N*-glycosylated proteins were involved in diverse functions in the infected RBCs such as complement reaction, coagulation cascades, immune and defense responses, heparin-binding, extracellular matrix–receptor interaction, cholesterol metabolism, and many disease-related pathways.

### Dynamic Protein Modifications in *P. falciparum* During the IDC

After invasion into an erythrocyte, the parasite needs to express novel proteins for energy production and creation of an environment for development and proliferation (25). Here, at the first step, we created an expression pattern clustering of modification sites of the parasite-derived proteins that showed significant changes over time (FDR < 0.01), and their functional associations with intraerythrocytic development were analyzed in depth.

**Fig. 2:**
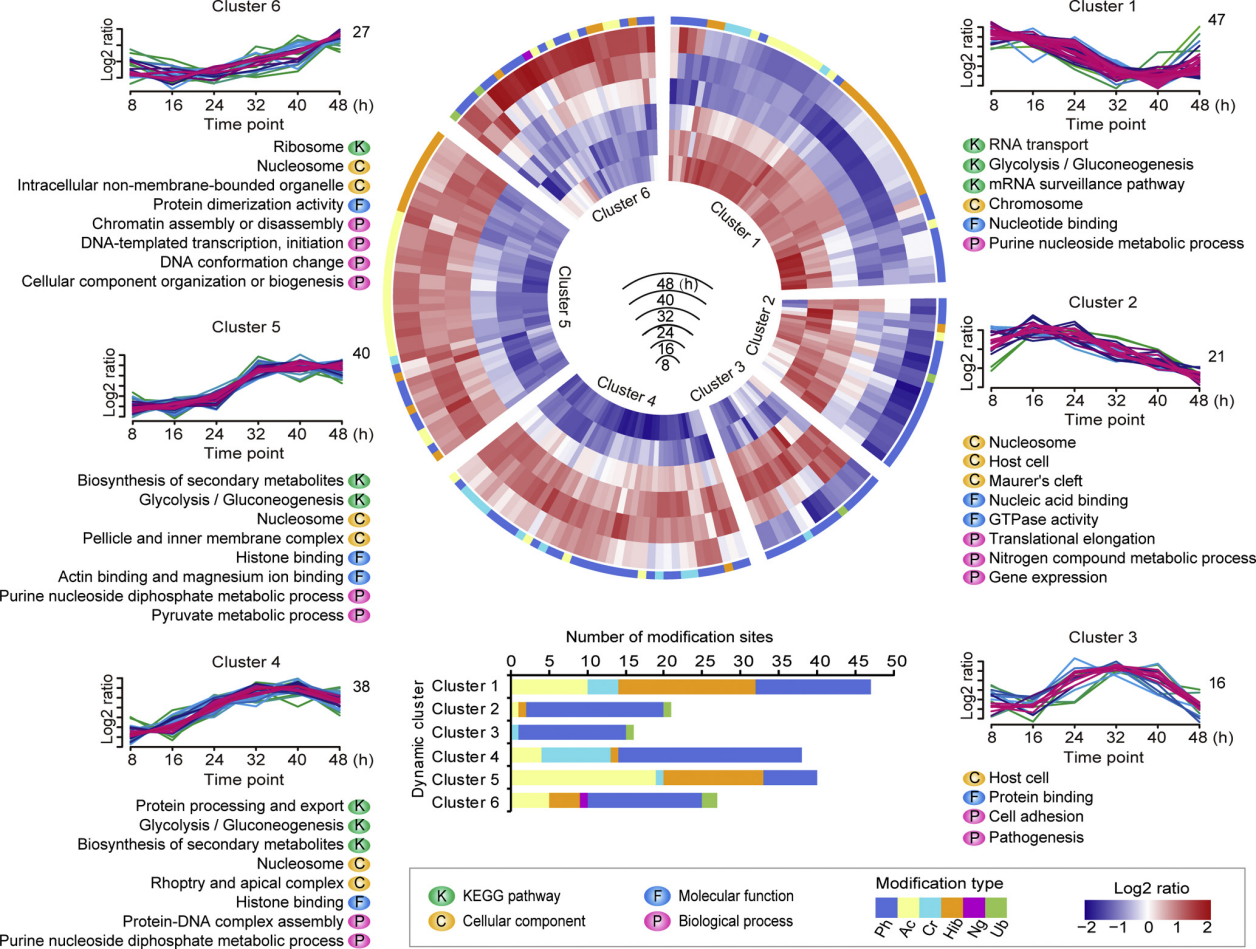
**Dynamic cluster heatmap of modification sites with significant changes (189 modification sites, FDR < 0.01) of *P. falciparum* proteins during the IDC**. The circular heatmap in the middle represents the abundance variation of each modification site over time from 8, 16, 24, 32, 40, and 48 h after invasion. The *redder* the color, the higher the abundance. The *bluer* the color, the lower the abundance. The six sections of the heatmap correspond to the six clusters based on the abundance trends of modification during the IDC, and the main function terms enriched in each cluster are displayed below the graphs. The function entries were enriched from KEGG pathways (represented by “K”) and GO terms, which were divided into three categories: cell components (represented by “C”), molecular function (represented by “F”), and biological process (represented by “P”). FDR, false discovery rate; GO, Gene Ontology; IDC, intraerythrocytic development cycle; KEGG, Kyoto Encyclopedia of Genes and Genomes.

The overall modification abundance of *P. falciparum* proteins in Cluster 1 included 47 modification sites associated with parasite maturation (Fig. 2). The proteins in this cluster were primarily involved in nucleotide binding, gene transcription, RNA transport, and mRNA surveillance; these were mainly modified by phosphorylation, acetylation, and 2-hydroxyisobutyrylation, and they were likely to promote gene activation during merozoite development and trophozoite maturation. For example, the transcriptional coactivator ADA2, an evolutionarily conserved component of histone acetyltransferase complexes involved in chromatin remodeling and transcriptional regulation in eukaryotes (26), was acetylated at three lysine sites, with the highest abundance at 8 h; the acetylation extent decreased with the development of the parasite (supplemental Dataset S26). The RNA helicase UAP56, an important factor in mRNA exportation and pre-mRNA splicing (27), was phosphorylated at S240 and showed high abundance at 8, 16, and 48 h (supplemental Dataset S26). Furthermore, the modifications of histone H2A (H2A-K19^ac^), H3 (H3-S29^ph^), (H3.v-S29^ph^), splicing factor (Q8IKE9-S244^ph^), translation initiation factor (EIF3D-S542^ph^), nucleic acid-binding protein (Q8I2Y5-S82^ph^, Q8IIT2-K199^cr^), nucleoside transporter (Q8IDM6-S16^ph^), and several ribosomal proteins (Q8ID50-K29^hib^ and Q8ID50-S57^ph^, C0H4A6-K83^hib^, and Q8ID32-K5^ac^ of ribosome biogenesis protein MRT4, Q8I655-S50^ph^ of ribosome-associated membrane protein RAMP4) were predominant in the early stages (supplemental Dataset S26, and to be further analyzed in a later section). This indicated that in the early stage of *P. falciparum* development, not only phosphorylation and acetylation (28) but also 2-hydroxyisobutyrylation may exert similar functions in gene activation, as was also observed in a recent study of *T. gondii* (23). In addition, the heat shock protein 70 (HSP70) family members were mainly modified by 2-hydroxyisobutyrylation apart from phosphorylation and crotonylation in Cluster 1 (supplemental Dataset S26); they are known to be important in protein homoeostasis and protein trafficking across the parasitophorous vacuole as chaperones and immunogens (29, 30).

The overall abundance of 21 modification sites of *P. falciparum* proteins in Cluster 2 showed a downward trend during the parasite development, similar to that of Cluster 1 (Fig. 2). The proteins in this cluster, which were predominantly phosphorylated, were components of the nucleosome and Maurer's cleft, nucleic acid binding, GTPase activity, nitrogen compound metabolic processes, translational elongation, and host–parasite interactions. Mature parasite–infected erythrocyte surface antigen (MESA), an erythrocyte cytoskeletal protein 4.1 binding protein (31), was phosphorylated at S355 with high abundance at 16 and 24 h (supplemental Dataset S26). Similarly, the lysine-rich membrane-associated *Plasmodium* helical interspersed subtelomeric (PHIST) b (LyMP), an RBC cytoskeletal-binding protein that adheres to proteins expressed on vascular endothelial cells resulting in sequestration (32), was also phosphorylated at S310 with high abundance (supplemental Dataset S26). ETRAMP 10.2, which participates in interactions with RBC membrane protein (33), was highly phosphorylated at S136 at the same time points as MESA and PHISTb (supplemental Dataset S26). In addition, extensive modification, including phosphorylation, acetylation, crotonylation, 2-hydroxyisobutyrylation, and ubiquitination of elongation factor 1α (*PF3D7_1357000*) associated with mRNA translation (34) was observed (supplemental Dataset S24). Furthermore, the translation elongation factor 2, responsible for the GTP-dependent translocation of the ribosome along mRNA and essential for protein synthesis (35) was modified by 2-hydroxyisobutyrylation at K302, with the highest abundance at 16 h. The three RNA-binding proteins Q8IJX8-S3, Q8IJZ3-S745, and Q8I2Y5-S24 were all phosphorylated with high abundance at 16 and 24 h (supplemental Dataset S26). Therefore, the functions of PTMs in proteins of Cluster 2 were mainly associated with translation regulation and parasite–host interaction.

Unlike Clusters 1 and 2, the overall modification abundance in proteins of Cluster 3 remained at a high level during the mature stage (24, 32, and 40 h) of the parasite. These proteins are predominantly involved in cell adhesion and pathogenesis and were mainly phosphorylated (Fig. 2). Cytoadherence-linked asexual protein 3.1 (CLAG 3.1), a protein that targets the rhoptry of *P. falciparum* (36), was phosphorylated at S1382 with high abundance at 24 and 32 h (supplemental Dataset S26). The *P. falciparum* protein GBP-130 (glycophorin-binding protein) was phosphorylated on T257 with high abundance at 24 and 32 h. Antigen 332 (Pf332), the largest known *P. falciparum* protein, is transported into the host RBC cytoplasm and is involved in adhesion, development, and cytoskeletal interaction (37); the protein was phosphorylated at S1379. The phosphorylation of T122 of merozoite surface protein 1 (MSP1) remained at high levels at 24, 32, and 40 h, a period of protein generation, accumulation, and translocation. Furthermore, the merozoite surface protein MSA180, an unknown-function protein in *P. falciparum*, was phosphorylated at T966 with high abundance at the same period of time (supplemental Dataset S26).

The overall PTM abundance of *P. falciparum* proteins in Cluster 4 with 38 members increased gradually from 8 to 40 h, then decreased afterward (Fig. 2). The proteins in this cluster contained components of rhoptry, apical complex, histone binding, protein-DNA complex assembly, protein processing and export, purine nucleoside diphosphate metabolic process, glycolysis, and gluconeogenesis. Rhoptry-associated membrane antigen, which is synthesized in early trophozoites and is associated with both rhoptry biogenesis and host cell invasion (38), was phosphorylated at S417, with the highest abundance at 40 h. The *P. falciparum* rhoptry protein RhopH3, playing essential roles in host cell invasion and nutrient uptake (39), was phosphorylated at S804, and the modification abundance was at the highest level at 40 h. Variation in modifications of heat shock proteins was identified in Cluster 4, and the protein types were more diverse, including HSP70 with crotonylation at K82, K436, and K539, and 2-hydroxyisobutyrylation at K268. BIP (a molecule homologous to HSP70) was identified with crotonylation at K575 and HSP110c with phosphorylation at S589 (supplemental Dataset S26). In addition, the modifications of sugar metabolic enzymes were identified in this cluster; those enzymes included phosphoglycerate mutase 1 with crotonylation at K106 and phosphoglycerate kinase with crotonylation at K143, and the modification abundance was high at 32 and 40 h (supplemental Dataset S26). Previous studies have indicated that parasites at the trophozoite stage are metabolically most active, and during this phase, there is an enrichment in transcription of genes encoding for amino acid, tRNA, ncRNA, DNA, pyruvate, glycolytic, and carbohydrate metabolic processes (40). Thus, PTMs likely facilitate the functions of these proteins.

The overall PTM abundance of *P. falciparum* proteins in Cluster 5 with 40 modification sites gradually increased from 8 to 32 h and tended to be stable from 32 to 48 h, the stage of generating multiple nuclei and forming progeny cells ready for egress (Fig. 2). The proteins in this cluster included components of nucleosome, pellicle, and inner membrane complex, and they mainly regulated histone binding, glycolysis, gluconeogenesis, pyruvate metabolic process, actin-binding, and magnesium binding. Up to 13 histone modification sites were identified in Cluster 5, and all were acetylated with high abundance at the last three time points (supplemental Dataset S26).

Furthermore, several sugar metabolic enzymes were also present in Cluster 5, and all were modified by 2-hydroxyisobutyrylation with high abundance at the last three time points (32, 40, and 48 h). These included pyruvate kinase (PK), GAPDH, fructose-bisphosphate aldolase, phosphoglycerate mutase 1, and phosphoglycerate kinase (supplemental Dataset S26). Previous studies indicated that lysine 2-hydroxyisobutyrylation can regulate glycolysis in response to nutritional cues (41). Our data also supported the hypothesis that 2-hydroxyisobutyrylation promoted the enzymatic activity for glycolysis in *P. falciparum*, particularly at the schizont stage. Remarkably, the components of the motor complex that drives entry of the invasive parasites were identified in Cluster 5, including glideosome-associated protein 45 with S103^ph^, myosin-A with S19^ph^, inner membrane complex protein IMC1c with two phosphorylation sites (S208^ph^, S267^ph^), and IMC1g with one phosphorylation site (S191^ph^) (supplemental Dataset S26). The high level of phosphorylation of these proteins at the last three time points indicates their essential roles in merozoite maturation.

The overall PTM abundance of *P. falciparum* proteins in Cluster 6 with 27 modification sites continued to increase over time, reaching the highest level at 48 h (Fig. 2). The proteins in this cluster included components of nucleosome and ribosome and proteins regulating chromatin assembly, DNA activities, protein dimerization activity, cellular component organization, and biogenesis (Fig. 2). Except for histones and their variants, the rest of the proteins were modified by phosphorylation, demonstrating once again that phosphorylation might play a critical role in the regulation of gene transcription and translation (supplemental Dataset S26).

Proteins of the motor complex also appeared in Cluster 6, including GAP40 with phosphorylation at S420, actin-1 (ACT1) with ubiquitination at K114 and K207, and IMC1c with phosphorylation at S146 and S266 (supplemental Dataset S26). Except for ACT1 with the highest ubiquitination abundance from 40 h onward, the rest of the proteins of the motor complex showed the highest abundance of phosphorylation before egress (supplemental Dataset S26). During merozoite invasion of erythrocytes, actin filaments lie beneath the inner side of the parasite plasma membrane (42). The interaction of the myosin with actin could then move the merozoite motor complex, with directionality provided by the orientation of the actin filaments. The role of ACT1 ubiquitination also clearly was not associated with proteolytic degradation. In contrast, both ubiquitination and phosphorylation may actively facilitate the assembly of the motor protein complex (43).

### Spontaneous Modification of Host Erythrocytic Proteins After Parasite Invasion

**Fig. 3:**
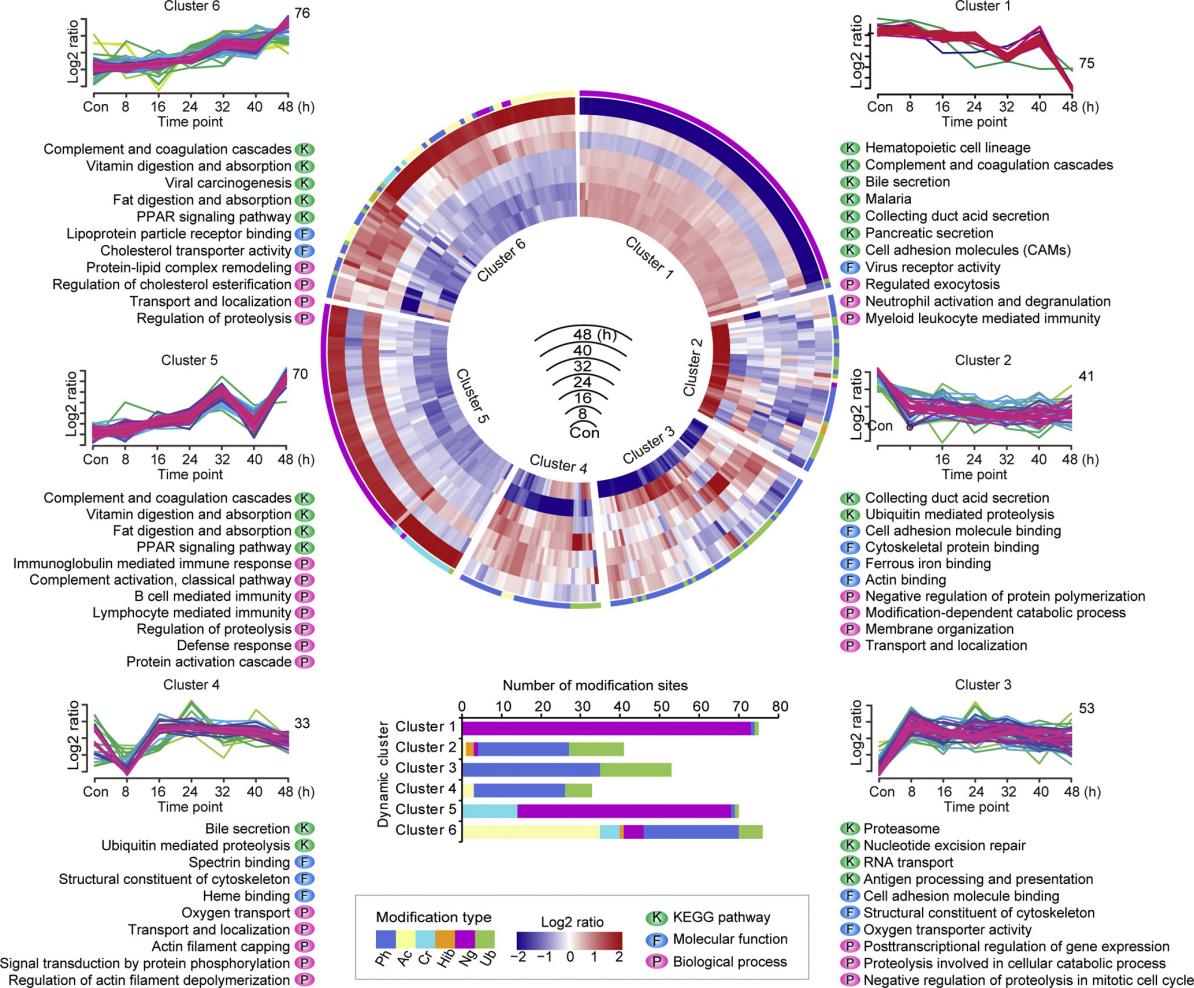
**Dynamic cluster heatmap of modification sites with significant changes (348 modification sites, FDR < 0.01) of RBC proteins**. The circular heatmap in the middle represents the abundance variation of each modification site of healthy RBC proteins and of *P. falciparum*−infected RBC proteins over time from 8, 16, 24, 32, 40, and 48 h after invasion. The *redder* the color, the higher the abundance. The *bluer* the color, the lower the abundance. The six sections of the heatmap correspond to the six clusters based on the abundance trends of modification during the IDC, and the main function terms enriched in each cluster are displayed below the graphs. The function entries were enriched from KEGG pathways (represented by “K”) and GO terms, which were divided into three categories: cell components (not shown in the figure), molecular function (represented by “F”), and biological process (represented by “P”). FDR, false discovery rate; GO, Gene Ontology; IDC, intraerythrocytic development cycle; KEGG, Kyoto Encyclopedia of Genes and Genomes.

Adaptation and evasion of immune clearance are critical for *P. falciparum* to develop and proliferate inside the blood circulation. After being invaded by the parasite, the host erythrocytes undergo profound structural and morphological changes, such as increased rigidity and adhesiveness, leading to sequestration in the microvasculature that not only enables the parasite to avoid spleen filtration but also results in organ dysfunction and severe malaria (44). Some studies have found that parasites could alter the activity of host proteins by PTMs (45, 46, 47). Here, the spontaneous modifications of the RBC proteins with significant variation in abundance at six time points were deeply characterized, taking normal erythrocytes as controls (FDR < 0.01) (Fig. 3; supplemental Dataset S27).

The overall PTM abundance of the erythrocyte proteins in Cluster 1 with 75 modification sites showed a slow downward trend with the development of *P. falciparum* inside the RBC (Fig. 3). The proteins in this cluster were mainly involved in cell adhesion, hematopoiesis, secretory activity, exocytosis, complement, and coagulation cascades, and they were predominantly modified by *N*-glycosylation. Many members of the cluster of differentiation (CD) antigen family, including CD35, CD44, CD47, CD55, CD58, CD59, and CD276, were extensively *N*-glycosylated with the development of the parasite inside the cell (supplemental Dataset S27). CD44 and CD55, which are widely distributed on RBCs, are both necessary for *P. falciparum* invasion (48, 49), and CD47 is linked to the membrane skeleton to enhance the deformability of red blood cells (50, 51). The complement receptor 1 (CR1), also known as CD35, which plays an important role in promoting invasion and rosette formation and clearance of immune complexes in malaria (5, 52), was glycosylated at four modification sites (N509, N578, N897, and N1908), with the lowest abundance at 48 h (supplemental Dataset S27, and to be further analyzed later). Apart from CD35, CD44, and CD55, we found several merozoite receptors, including basigin, intercellular adhesion molecule 4, and glycophorin-A (GPA), that were extensively *N*-glycosylated in the early stages of parasite development (supplemental Dataset S27). Because *P. falciparum* lacks sufficient glycosylation enzymes, the modifications of erythrocytic proteins were likely triggered by signaling during parasite invasion (53).

The PTM abundance of erythrocyte proteins in Cluster 2 with 41 modification sites, in contrast to those of other proteins, remained significantly lower in the iRBC proteins than in normal RBCs (Fig. 3). The proteins in this cluster were mainly involved in ubiquitin-mediated proteolysis, modification-dependent catabolic processes, protein binding, ferrous iron-binding, membrane organization, and protein polymerization, transport, and localization. The ubiquitination of neural precursor cells expressed developmentally downregulated protein 8 (NEDD8), which is conjugated to target proteins or lipids to regulate their activity, stability, subcellular localization, or macromolecular interactions (54), was much lower in iRBCs than in RBC controls (supplemental Dataset S27). Previous studies have shown that ubiquitin chains linked to K48 led to degradation of the substrate via the 26S proteasome (13). The deubiquitination and higher quantity of NEDD8 compared with that of normal RBCs postparasite invasion indicated that NEDD8 and its partner proteins were necessary for cell stability and the intracellular development of the *P. falciparum* (supplemental Datasets S24 and S27). Similarly, the autophagy-related protein 2 was dephosphorylated at S403 in iRBCs compared with that of RBCs (supplemental Dataset S27), suggesting that the autophagy signaling pathway mediated by phosphorylation was suppressed by *P. falciparum* after invasion. Furthermore, the ubiquitination of many erythrocyte cytoskeleton proteins such as spectrin, actin, cytoplasmic 2 (ACTG1), ankyrin-1, band 3 anion transport protein (SLC4A1), and dematin (DMTN) that contribute to the deformability and stability of erythrocytes was also lower in iRBCs than in RBCs (supplemental Dataset S27). Meanwhile, the quantity of most erythrocyte cytoskeleton proteins remained higher in iRBCs than in RBCs (supplemental Dataset S27), suggesting that the RBC proteins were selectively protected for the benefit of parasite development.

The PTM abundance of the infected erythrocyte proteins in Cluster 3 with 53 modification sites was significantly higher than that of the RBC control (Fig. 3). The prominent components in this cluster were proteolytic-related proteins of the ubiquitin-associated proteasome system (UPS), including ubiquitin-activating enzyme E1, ubiquitin conjugating enzyme E2, ubiquitin-protein ligase E3, 26S proteasome and deubiquitinating enzymes, ubiquitin-60S ribosomal protein L40 (UBA52), proteasome activator complex subunit 1, proteasome subunit alpha type-1 (PSMA1), PSMA3, PSMA4, and PSMD2. These UPS-associated proteins were ubiquitinated and phosphorylated predominantly in the trophozoite stage (24, 32 h) except for RNF123, of which the modification was higher in the early ring stage (8 h), and the modification of PSMD2 in the schizont stage (supplemental Dataset S27). Thus, it is likely that the parasite promoted the enzymatic activity through ubiquitination and phosphorylation. In addition, antioxidant proteins with modifications were also detected in Cluster 3, including peroxiredoxin 6 (PRDX6) and thioredoxin-related transmembrane protein 1 (TMX1). Peroxiredoxin 6 was predominantly phosphorylated on T177 at 8 h. TMX1, which plays a major role in host defense under oxidative stress (55), was phosphorylated at S270, with the highest abundance at 8, 24, and 32 h and the lowest abundance at 48 h (supplemental Dataset S27).

The erythrocyte-derived proteins with 33 modification sites in Cluster 4 were mainly involved in ubiquitin-mediated proteolysis, structural constituents of the cytoskeleton, actin filament capping and depolymerization, spectrin binding, heme binding, oxygen transport, signal transduction, transport, and localization (Fig. 3). Similar to Cluster 2 and Cluster 3, many cytoskeletal proteins and cell degradation–related proteins were found in Cluster 4, and these were mainly modified by phosphorylation and ubiquitination (Fig. 3; supplemental Dataset S27). Cytoskeletal proteins included tubulin beta-4B chain (TUBB4B) with ubiquitination at K58; DMTN with phosphorylation at S85; S156, S152, spectrin alpha chain, and erythrocytic 1 (SPTA1) with phosphorylation at S1284; beta-adducin (ADD2) with phosphorylation at S455; S592, and S600; and ankyrin-1 with phosphorylation at S1872 and S834. The ubiquitination abundance of TUBB4B-K58 showed low levels at 8, 16, 24, and 32 h and the highest level at 40 h, suggesting that TUBB4B was likely degraded in the schizont stage via the UPS pathway. Furthermore, hemoglobins were acetylated (HBB-K62^ac^, HBG1-K62^ac^, HBD-K62^ac^), with the lowest modification abundance at 8 h and the highest levels at 32 and 40 h (supplemental Dataset S27), suggesting that acetylation may also be involved in the hemoglobin degradation.

*P. falciparum* is unable to synthesize purines *de novo* and must obtain them from the host, and the human nucleoside transporter is one of the main mediators for *P. falciparum* in uptake of purines into iRBCs (56). Here, we found the ubiquitination abundance on K255 of the equilibrative nucleoside transporter 1 (SLC29A1) was low at 8, 16, and 48 h and high at 24 h (supplemental Dataset S27). Furthermore, the two members of SLC in the efflux transport of organic anions, namely, solute carrier family 2 (SLC2A1) and solute carrier family 43 member 3 (SLC43A3), were deubiquitinated p.i. compared with those of the RBC control (supplemental Dataset S27). Consequently, the quantities of the two functional proteins reached the highest levels at 16 h and remained much higher than in the RBCs (supplemental Dataset S27), possibly because of the need for nutrient uptake and transport into the parasite via erythrocytes, especially in the ring stage and early trophozoite stage.

The overall PTM abundance of RBC proteins in Cluster 5 with 70 modification sites showed an upward trend from 8 h to 32 h (Fig. 3). The proteins in this cluster are mainly involved in complement and coagulation cascades, immune and defense responses, the PPAR signaling pathway, protein activation cascade, proteolysis, and digestion and absorption of vitamins and fats. These proteins were mainly modified by *N*-glycosylation and crotonylation. For example, C3 with crotonylation at K1526, C4A with phosphorylation at S1177, C4B with glycosylation at N1328, C8A with glycosylation at N437, CFH with glycosylation at N1029 and N911, and C4b-binding protein beta chain (C4BPB) with glycosylation at N64 and N71 (supplemental Dataset S27). The coagulation cascade, a complex process of proteolytic reactions, is closely associated with the complement system and depends on multiple coagulation factors (Factor I∼XIII) (57). Here, glycosylation of prothrombin (N143) and coagulation factor XI (N126 and N450) were the highest at 48 h, similar to the results for Cluster 1. We also found a CD antigen, CD14, that was glycosylated at N151 predominantly at 48 h (supplemental Dataset S27). CD14 is a multifunctional receptor expressed on many cell types, and it has been shown to mediate the immune response, resulting in the activation of an inflammatory cascade (58).

We identified several apolipoproteins in Cluster 5 with the highest PTM abundance at 48 h, including APOA1 (APOA1-K157^cr^), apolipoprotein E (APOE, APOE-K175^cr^, APOE-K260^cr^), apolipoprotein B-100 (APOB B-100, APOB-K2139^cr^, APOB-K4485^cr^), and glycosylation of APOB (N1523^gly^, N3465^gly^) (Fig. 3; supplemental Dataset S27). Apolipoproteins bind, dissolve, and transport hydrophobic lipids in the blood (59). APOA1 levels are positively correlated with hemoglobin levels in malaria-infected primiparas, and low levels of APOA1 are associated with anemia, inflammatory deterioration, and poor prognosis in primiparous pregnant women during malaria infection (60). *P. falciparum* undergoes a rapid proliferation fueled by *de novo* synthesis and acquisition of host cell lipids (61). Thus, crotonylation and glycosylation may facilitate the lipid transportation activity of apolipoproteins.

The PTM abundance of proteins in Cluster 6 with 76 modification sites continued to increase over time from 8 h, reaching the highest level at 48 h during the IDC (Fig. 3). Proteins in this cluster were mainly involved in the regulation of complement and coagulation cascades, proteolysis, cholesterol transporter activity, fat digestion and absorption, protein-lipid complex remodeling, vitamin digestion and absorption, and the PPAR signaling pathway. Similar to Cluster 5, complement proteins were still present in Cluster 6, but the modification was shifted from *N*-glycosylation to acylation (Fig. 3; supplemental Dataset S27). There were three crotonylation sites (K678, K1050, and K1325) and two acetylation sites (K1050 and K1203) on C3 and one acetylation site (K732) on CFB. The modification abundance was the highest at 48 h. Furthermore, phosphorylation of plasminogen and antithrombin-III (SERPINC1) increased with parasite development. The transformation from plasminogen to plasmin is the central reaction of the fibrinolytic system that may facilitate merozoite egress (62).

### Dynamic Modification of Histones and Gene Regulation During the IDC

*P. falciparum* displays diverse features of its epigenome, such as the absence of linker histone H1 (63) and RNA interference machinery (64), low abundance of DNA methylation (65), and the presence of unusual histone variants with a unique set of modifications (66, 67). To date, there has been extensive research on PTMs of *P. falciparum*, but investigations of dynamic modification over the time course of *P. falciparum* development are relatively fewer, except for histone dynamic modifications (18, 68, 69). Unlike the majority of higher eukaryotes, *P. falciparum* chromatin is predominantly in a euchromatic state with only a few heterochromatic islands marked by trimethylation of the ninth lysine (K) of histone 3 (H3K9^me3^) (18, 70, 71). Euchromatic upstream regulatory regions of most genes are typically associated with the presence of the histone variants H2A.z and H2Bv, H3K9^ac^, and H3K4^me3^ (28). Acetylation of H3K9 at promoter regions correlates with the gene transcriptional status, whereas H3K4^me3^ appears to promote stage-specific regulation of gene expression (72, 73, 74).

**Fig. 4:**
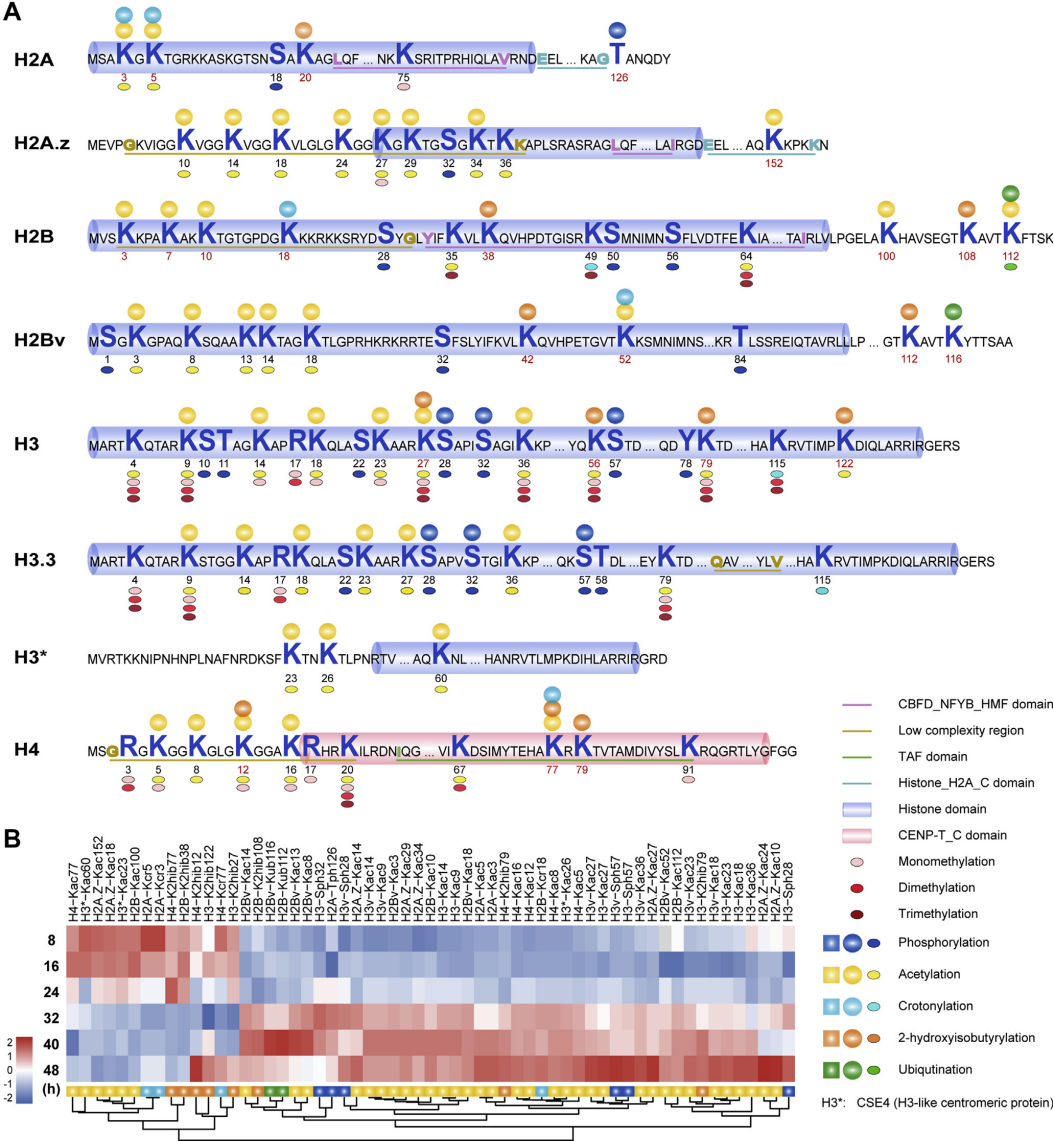
**PTMs of histones (H2A, H2B, H3, and H4) and histone variants (H2A.z, H2B variant, H3 variant, and H3-like centromeric protein CSE4)**. *A*, distribution diagram of modification sites of histones and histone variants. The conserved domains, including the histone domain, Histone_H2A_C domain, CBFD_NFYB_HMF domain, CENP-T_C domain, low complexity region, and TAF domain, are marked with colored lines. The PTMs identified in this study are presented on the corresponding amino acids, and novel PTMs identified are indicated with circular spheroids with gradient colors. PTMs identified earlier are represented underneath the corresponding amino acids in small oblate spheroids with even colors. Methylation (including monomethylation, dimethylation, and trimethylation) identified earlier is also displayed in addition to the modifications involved in this research. *B*, dynamic cluster heatmap of quantifiable modification sites on histones and histone variants. Log2 geometric means of normalized expression values (from the signal intensity of MS/MS) of the three repetitions were used to indicate the abundance of the modification site at a specific time during the IDC. The *redder* the color, the higher the abundance. The *bluer* the color, the lower the abundance. The middle color is *white*. The modification sites are named “histone name-amino acid-site number-PTM type.” (Notes: In most PTM-omics studies, the number of amino acid positions takes the starting amino acid methionine [M] as Position 1, so “M” was marked as Position 1 in the full text of this article. However, the number of sites of histones takes the next amino acid “M” as Position 1 in the existing histone modification research on *P. falciparum*. To facilitate the comparison and discussion with the existing literature, we subtracted one from the number of all histone sites, which was consistent with the previous studies on histone modifications in *P. falciparum*.) IDC, intraerythrocytic development cycle; PTMs, posttranslational modifications.

Here, a much more comprehensive histone PTM map of *P. falciparum* was completed (Fig. 4*A*). This study covered most of the modification sites discovered, and 27 new modification sites were identified; these were H2AK3^cr^, H2AK5^cr^, H2AK20^hib^, H2AT126^ph^, H2A.zK152^ac^, H2BK3^ac^, H2BK7^ac^, H2BK10^ac^, H2BK18^cr^, H2BK38^hib^, H2BK100^ac^, H2BK108^hib^, H2BK112^ac^, H2BvK42^hib^, H2BvK52^ac^, H2BvK52^cr^, H2BvK112^hib^, H2BvK116^ub^, H3K27^hib^, H3K56^hib^, H3K79^hib^, H3K122^hib^, H4K12^hib^, H4K77^ac^, H4K77^cr^, H4K77^hib^, and H4K79^hib^. The *P. falciparum* histones (H2A, H2B, H3, and H4) and histone variants (H2A.z, H2Bv, H3v, and H3∗) underwent extensive modification, and the modification level changed significantly over time (Fig. 4; supplemental Dataset S28). Most of the histone PTMs appeared at the N termini, which were mainly acetylated, followed to a lesser extent by phosphorylation, crotonylation, and 2-hydroxyisobutyrylation (Fig. 4*A*). Some modifications of histones appeared at the C termini, including 2-hydroxyisobutyrylation, acetylation, ubiquitination, phosphorylation, and crotonylation. A few modifications also occurred in the central areas (Fig. 4*A*). The two histone variants, H2A.z and H3∗ (CSE4, H3-like centromeric protein), were only acetylated. Ubiquitination was identified in H2B and H2Bv, named H2BK112^ub^ and H2BvK116^ub^ (Fig. 4*A*), and mainly occurred at 32, 40, and 48 h of IDC (Fig. 4*B*). Phosphorylation predominantly occurred on both H3 (H3S22, H3S28, H3S32, and H3S57) and the H3 variants (H3vS22, H3vS28, H3vS32, and H3vS57) (Fig. 4*A*). Overall, the abundance of most modification sites of histones and histone variants was at high levels at the last three time points (32, 40, and 48 h) (Fig. 4*B*), probably because the nucleus began to divide from about 32 h, resulting in the increasing of chromosomal activity.

**Fig. 5:**
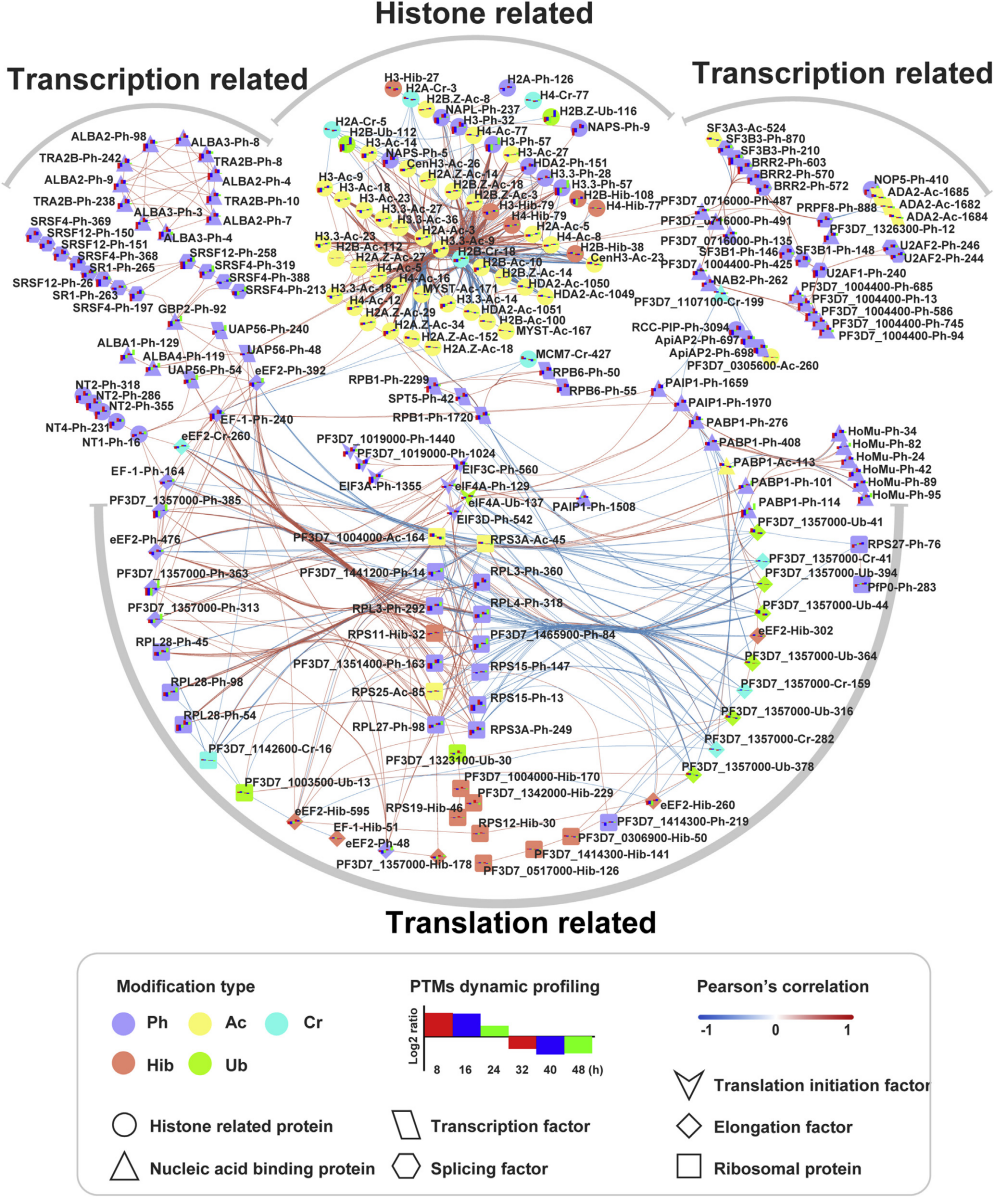
**Networks of modified proteins associated with gene regulation and expression**. Histone-related proteins are shown in the middle of the top area. The left and right sides are transcription-related proteins, including the transcription factors, splicing factors, and elongation factors. The elliptical region below indicates translation-related proteins, including the translation factors and ribosomal proteins in the middle and the elongation factors on both sides. *Small triangles* represent nucleic acid-binding proteins distributed in multiple regions. The colors of the lines indicate Pearson's correlation (Pearson's correlation > 0.9). The *red line* represents a positive correlation, and the *blue line* represents a negative correlation. Modification type and PTMs dynamic profiling are shown in the figure. PTMs, posttranslational modifications.

Accumulating evidence suggests that the chromatin of *P. falciparum* is highly organized, and this structure provides an epigenetic mechanism for transcriptional regulation (75). Here, we found that the transcription-associated proteins were mainly modified by phosphorylation, with lesser amounts of acetylation and ubiquitination (Fig. 5). The parasite-specific transcription factor PfAP2-I (*PF3D7_1007700*), a potential new antimalarial therapeutic target, is responsible for regulating the expression of genes involved in RBC invasion (76). Two phosphorylation sites were identified in this protein, PfAP2-I-S913^ph^ with high abundance at 8 and 48 h and PfAP2-I-T942^ph^ with high abundance at the mature trophozoite stage (supplemental Datasets S24 and S29); the turnover of phosphorylation sites thus might control the timing of the regulatory function of the transcription factor. The translation initiation factors, such as eIF2A, EIF3A, EIF3C, EIF3D, EIF3I, eIF4A, EIF4A3, EIF5, and EIF3M, were mainly phosphorylated, whereas eIF4A and EIF4A3 were also ubiquitinated, and EIF3M was 2-hydroxyisobutyrylated (Fig. 5, supplemental Dataset S24). Some of the modifications occurred in the ring stage, for example, EIF3C-S560^Ph^, EIF3C-S564^Ph^, EIF3D-S542^Ph^, eIF4A-T129^Ph^, and -K39^Ub^, and some occurred in the late trophozoites and schizont stages, such as eIF2A-S1024^Ph^, eIF2A-S1440^Ph^, and eIF4A-K137^Ub^ (supplemental Datasets S24 and S29). Of the three members of the eIF2α kinase family (PfeIK1, eIK2, and PfPK4), PfeIK1 regulates nutritional stress response during asexual growth of *P. falciparum*; eIK2 plays a major role in maintaining translational silencing in sporozoites; and activation of PfPK4 leads to the arrest of global protein synthesis not only during ontogeny of daughter merozoites but also in mature gametocytes (22). We identified phosphorylation at S2345 of PfPK4, and the modification abundance reached high levels at 8, 40, and 48 h (supplemental Dataset S24). In addition, the modification types of the elongation factors and the ribosomal proteins were more diverse, with prominent phosphorylation, 2-hydroxyisobutyrylation, and lesser amounts of ubiquitination, crotonylation, and acetylation (Fig. 5; supplemental Dataset S29). This indicated that the translation initiation of *P. falciparum* proteins was mainly regulated by phosphorylation, but subsequent processes were coregulated by other modifications.

### PTM-omics of Parasite-Derived Pathogenic Factors During the IDC

**Fig. 6:**
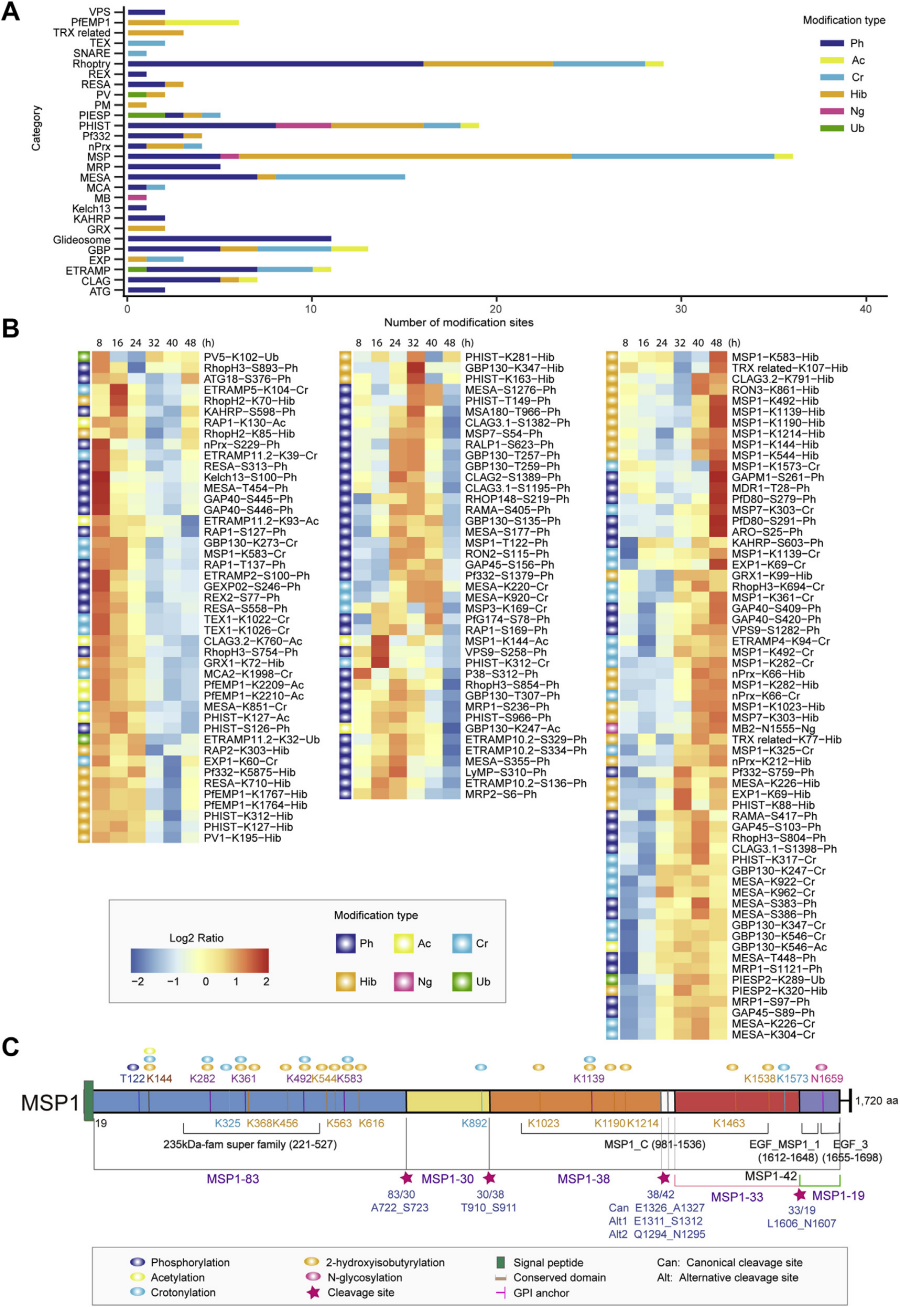
**The posttranslational modifications of pathogenesis-related proteins in *P. falciparum***. *A*, the bar chart shows the number of modification sites in each pathogenesis-related parasite protein family. *B*, dynamic cluster heatmap of modification sites of the proteins during the IDC. The heatmap represents the abundance variation of each modification site over time. The modification sites are named “protein name-amino acid type-site number-PTM type,” such as “MSP1−K325−Cr.” *C*, the modification of MSP1. The conserved domains and modification sites of MSP1 are shown in the figure. The conserved domains contain the 235 kDa-fam superfamily domain, MSP1_C domain, EGF_MSP1_1 domain, and EGF_3 domain. IDC, intraerythrocytic development cycle; MSP 1, merozoite surface protein 1; PTM, posttranslational modifications.

The MSP family-associated host cell invasion contained the largest number of modified sites of phosphorylation, acetylation, crotonylation, 2-hydroxyisobutyrylation, and *N*-glycosylation, and the PTM predominantly occurred at the later stages of parasite development (Fig. 6*B*), indicating that PTMs may actively regulate the function of this critical protein family. In *P. falciparum*, MSP1 is a polymorphic protein that interacts with other peripheral merozoite surface proteins such as MSP3, MSP7, serine repeat antigen 4, and serine repeat antigen 5 to form a large complex (77). MSP1, MSP3, and MSP7 all undergo proteolytic processing during the schizont stage or at the tight junction between the invading merozoite and erythrocyte (77, 78, 79). We identified one crotonylated site of MSP3 at K169 with the highest abundance at 40 h; three modification sites of MSP7, including phosphorylated S54 with the lowest abundance at 48 h; and 2-hydroxyisobutyrylated and crotonylated K303 with the highest abundance at 48 h (Fig. 6*B*; supplemental Dataset S30). Compared with those on MSP3 and MSP7, PTMs on MSP1 were much more prevalent. It is known that proteolytic maturation of MSP1 is important for parasite viability because of its binding activity to erythrocyte spectrin, which critically regulates parasite egress from *P. falciparum*–infected red blood cells (pRBCs) (80). Surprisingly, we identified 28 modifications on 21 residues of MSP1, with 16 2-hydroxyisobutyrylation sites, nine crotonylation sites, one phosphorylated site, one acetylation site, and one *N*-glycosylation site (Fig. 6*C*; supplemental Dataset S30). Similar to that of MSP7 (MSP7-S54^ph^), phosphorylation occurred at the N terminus of MSP1 (MSP1-T122^ph^), and the modification abundance showed the lowest at 48 h (Fig. 6, *B*–*C*). Otherwise, many modification sites on MSP1 were located in the conserved domains, such as K282^hib^, K282^cr^, K325^cr^, K361^hib^, K361^cr^, K368^hib^, K456^hib^, K492^hib^, and K492^cr^ in the fam superfamily domain; K1023^hib^, K1139^hib^, K1139^cr^, K1190^hib^, K1214^hib^, and K1463^hib^ in the C domain; and N1659^gly^ in the EGF_3 domain (Fig. 6*C*), indicating that the modifications may critically regulate functions such as proteolytic processing and molecular binding. The modifications K1463^hib^, K1538^hib^, K1573^cr^, and N1659^gly^, with the highest levels at 48 h, were located in the MSP1-42 fragment. The level of glycosylation (N1659) in MSP1_19_, the only fragment of MSP1 that was carried into erythrocytes with the parasite, reached the highest at 48 h (Fig. 6, *B*–*C*).

During *Plasmodium* infection, the host immune system along with the inflammatory response is activated to eliminate the parasites (5). To escape the attack, the surface of pRBCs changes significantly to avoid immune recognition and to adhere to the vascular cells for escaping spleen-mediated filtration (4). Cytoadherence of the iRBCs is conferred by a high-molecular-weight protein, *P. falciparum* erythrocyte membrane protein 1 (PfEMP1), that is encoded by approximately 60 *var* genes per haploid genome. The parasite selects only one PfEMP1 to be expressed and transported to the pRBC surface (81). PfEMP1 expression starts 4 h p.i.; it is transported to and stored in Maurer's clefts and does not appear on the pRBC surface until 16 h. We identified three members of the PfEMP1 family, Q8I520 (*PF3D7_1240400*) with 2-hydroxyisobutyrylation at K1764, K1767; A0A143ZZY8 (*PF3D7_0833500*) with acetylation at K385, K390; and Q8IHM0 (*PF3D7_1150400*) with acetylation at K2209, K2210. The modification abundance was maintained at a high level from the early to the mature trophozoite stage (8, 16, and 24 h) but decreased from 32 h and reached the lowest level at 40 h. The modification sites were located in both extracellular and intracellular regions of the molecule. We did not observe phosphorylation in these PfEMP1s as reported recently (82), probably because of the lack of expression of the chondroitin sulfate A-binding variant in our parasites.

In addition to MSP and PfEMP1, we also observed heavy modification occurring in other parasite-derived proteins. MESA was modified with 2-hydroxyisobutyrylation at K226; crotonylation at K220, K226, K304, K851, K920, K922, and K962; and phosphorylation at S177, S355, S383, S386, S1276, T448, and T454. Ring-infected erythrocyte surface antigen (RESA) was 2-hydroxyisobutyrylated at K710 and phosphorylated at S313 and S558. The largest known protein (Pf332), transported into the host cell cytoplasm and probably involved in adhesion and cytoskeletal interaction (37), was identified as being phosphorylated at S2346 in previous studies (17). We found that this protein was 2-hydroxyisobutyrylated at K5875 and phosphorylated at S759, S1379, and S2583 (Fig. 6*B*; supplemental Dataset S30). Furthermore, the exported proteins, PHIST family with 89 members, underwent extensive modifications (Fig. 6, *A*–*B*; supplemental Fig. S4; supplemental Dataset S30). Based on the presence and positions of several conserved tryptophan residues, the PHIST protein family has been divided into three subgroups: PHISTa with 26 members, PHISTb with 24 members, and PHISTc with 18 members (83). PHIST proteins are central to host cell remodeling, but despite their obvious importance in pathology, PHIST proteins seem to be understudied (84). *PF3D7_0424600* (PHISTb) has been found to be phosphorylated at T124 in previous studies (17). Here, we identified 22 modification sites matched to 15 proteins in the PHIST family, and the sites involved phosphorylation, 2-hydroxyisobutyrylation, crotonylation, *N*-glycosylation, and acetylation (supplemental Fig. S4; supplemental Dataset S30). *PF3D7_0402000* was 2-hydroxyisobutyrylated at K163 with the highest modification abundance at 32 h (supplemental Fig. S4; supplemental Dataset S30). PHISTb proteins characterized were localized at and might interact with the host cell cytoskeleton. Phosphorylation widely existed in PHISTb, and the phosphorylation levels of most members had significant fluctuations at 8 or 48 h, suggesting that PHISTb might be involved in phosphorylation-mediated signal transduction and parasite–host interactions during the release or invasion of the parasite. We also identified 2-hydroxyisobutyrylation and crotonylation in PHISTb, suggesting that these two modifications might be involved in host cytoskeleton remodeling (supplemental Fig. S4; supplemental Dataset S30). RESA (*PF3D7_0102200*) is one of the seven members of the PHISTb-DnaJ subgroup. Previous study has shown that at the cytoskeleton, RESA is phosphorylated and interacts with spectrin (85). We further found that the protein was 2-hydroxyisobutyrylated at K710, in addition to the phosphorylation at S313 and S558, which strongly suggested that 2-hydroxyisobutyrylation might be involved in the interaction between the parasite and the host cytoskeleton. Several PHISTc proteins have been found in structures such as Maurer's clefts and exosome-like vesicles and are thought to be involved in protein trafficking (84). There is evidence for PHISTc protein *PF3D7_0936800* to also be localized at the host cell membrane and that the protein interacts with the acidic C-terminal segment domain of PfEMP1, albeit at a level much weaker than LyMP (PHISTb, *PF3D7_0532400*). We identified 2-hydroxyisobutyrylation at K281 with high modification abundance at 16, 32, and 48 h (supplemental Fig. S4; supplemental Dataset S30). In addition, we identified *N*-glycosylation and acetylation in PHISTc, but not in PHISTa or PHISTb, indicating that the function of PHISTc might be relatively more diverse (supplemental Fig. S4).

### PTMomics of Key Receptors on the RBC Surface Associated With Parasite Invasion and Pathogenesis

**Fig. 7:**
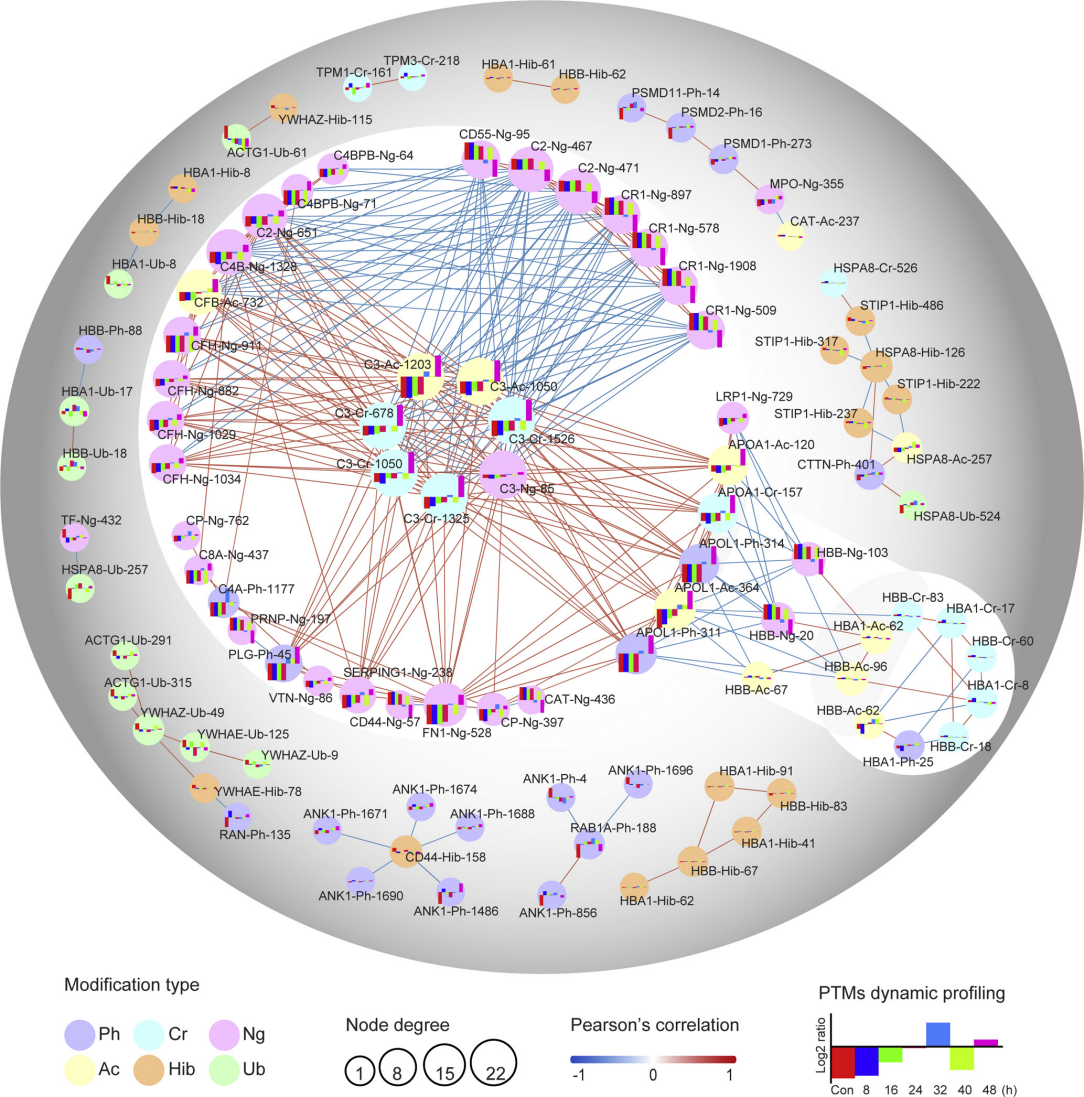
**Protein–protein interaction network of disease-related proteins of RBCs**. The proteins in the interaction network contain complement factors (C2, C3, C4A, C4B, C4BPB, and C8A), complement receptor CR1/CD35, CD molecules (CD44, CD55), hemoglobin (HBA, HBB), and apolipoproteins (APOA1, APOL1). The size of the circle represents the node degree (depending on the number of connecting lines), and the colors of the lines indicate Pearson's correlation (Pearson's correlation >0.8). The *red line* represents a positive correlation, and the *blue line* represents a negative correlation. The stronger the correlation, the darker the color. Histograms of dynamic abundance variation of each modification site are displayed in the circles. PTMs, posttranslational modifications.

The RBC receptors of *P. falciparum* ligands were actively modified with significant PTM changes (FDR < 0.01) during IDC. Here, an interaction network (Pearson's correlation > 0.8) of the proteins based on the modification sites (Fig. 7; supplemental Dataset S31) was created, and the proteins positioned at the key points were complement factors and receptors, CD molecules, and apolipoproteins (Fig. 7), all of which have been associated with *P. falciparum* invasion, immune response, erythrocyte deformability, anemia, and lipid metabolism.

The erythrocyte invasion pathway of *P. falciparum* can be either sialic acid–dependent or sialic acid–independent (5). The receptors of the sialic acid–dependent pathway include GPA, GPB, and GPC, which are glycophorins recognized by the parasite EBA family members (86, 87, 88, 89, 90, 91). The receptors of sialic acid–independent invasion are CR1 (52), CD147/Basigin (92), Kx (90), CD44 (93), and CD55 (49). It is generally believed that all human glycophorins contain similar *O*-glycans, and GPA and GPC also contain *N*-glycosidic chains at N26 and N8 residues, respectively (89). Here, we identified an *N*-glycosylation site in GPA at N45 with the lowest abundance at 48 h, and we also identified two ubiquitination sites at K120 and K126, with the highest levels at 16 h. Variations in the modification of GPC during IDC were observed, including 2-hydroxyisobutyrylation and crotonylation on K88 with the highest abundance at 48 h, crotonylation on K97 with the highest abundance at 40 h, phosphorylation on T90 with the lowest abundance at 8 h, and phosphorylation on S104 with the highest abundance at 32 h (supplemental Dataset S24). In this aspect, the parasite-modulated GPA phosphorylation cascade was clearly present (94).

We identified five *N*-glycosylation sites on CR1/CD35 (N447, N509, N578, N897, and N1908) (supplemental Dataset S24), and four showed significant PTM abundance variation, except for CR1-N447^gly^ (Fig. 7; supplemental Dataset S31). The modification levels of each site of CR1 had similar trends, reaching the lowest level at 48 h (supplemental Dataset S24). As mentioned earlier, many CD molecules, such as CD14, CD44, CD47, CD55, CD58, CD59, and CD276, were regulated by *N*-glycosylation (supplemental Dataset S24). In contrast, the modifications of most of the complement factors, including C1–9, CFB, CFH, CFI, and C4BP, were diverse, including *N*-glycosylation, acetylation, crotonylation, and phosphorylation (supplemental Dataset S24).

The PTM abundances of the seven modification sites on C3 (C3-K678^cr^, C3-N85^gly^, C3-K1203^ac^, C3-K1325^cr^, C3-K1050^cr^, C3-K1050^ac^, and C3-K1526^cr^) were positively correlated with one another; meanwhile, the abundance of the four *N*-glycosylation sites on CFH (CFH-N1034^gly^, CFH-N882^gly^, CFH-N1029^gly^, and CFH-N911^gly^) and the acetylation on CFB (CFB-K732^ac^) were also positively correlated with that of C3 (Fig. 7; supplemental Dataset S31). However, the abundance of *N*-glycosylation of CR1 (N509^gly^, N578^gly^, N897^gly^, and N1908^gly^) was negatively correlated with those of C3, CFB, and CFH. The abundance of *N*-glycosylation of CD55 (CD55-N95^gly^) was positively correlated with that of CR1 and negatively correlated with that of C3 (Fig. 7). Because the PTM abundance at a specific time reflected the activeness of the proteins at the time because of PTM regulation being a fast and efficient way to regulate life activities (9), the negative correlation between the PTM abundance of the two receptor groups indicated that PTM regulation was actively manipulated by the parasite during host immune attack. In addition, apolipoproteins could interact with C3 and hemoglobin HBB, and the abundance of the modification sites (APOA1-K120^ac^, APOA1-K157^cr^, APOL1-S314^ph^, APOL1-K364^ac^, and APOL1-S311^ph^) of apolipoproteins was positively correlated with that of C3 and negatively correlated with that of HBB (Fig. 7).

### PTM-omics of Proteins in the Key Metabolic Pathways of *P. falciparum*

During the erythrocytic stage, *P. falciparum* relies principally on anaerobic glycolysis for energy production, and the enzymes of parasite glycolysis were found modified by phosphorylation, acetylation, crotonylation, 2-hydroxyisobutyrylation, and ubiquitination, and the hypoxanthine-guanine phosphoribosyltransferase (HPRT) in the purine salvage pathway was extensively modified by acylation (acetylation, crotonylation, and 2-hydroxyisobutyrylation) (supplemental Fig. S5). Phosphofructokinase and PK are the rate-limiting enzymes in the glycolytic pathway (95), and these were modified by 2-hydroxyisobutyrylation, crotonylation, acetylation, and phosphorylation, and PK-K38^ac^ and PK-K477^hib^ predominantly occurred at the early trophozoite stage; PK-K116^hib/cr^, PK-K121^cr^, and PK-S461^ph^ had high abundance at the later developmental stages (supplemental Dataset S24). Interestingly, all modifications occurred in the conserved domain of phosphofructokinase (supplemental Fig. S5) and not in the active site of the enzyme, suggesting that, unlike in *Trypanosoma brucei* (96), the PTMs are mainly involved in the structural homeostasis of the enzymes in *P. falciparum*.

We did not observe any modification of adenine phosphoribosyltransferase, but we identified 2-hydroxyisobutyrylation, acetylation, and crotonylation on HPRT, including HPRT-K103^hib^, HPRT-K37^hib^, and HPRT-K223^ac^ with high abundance at early developmental stages and highly abundant HPRT-K62^cr^ at the later developmental stages (supplemental Dataset S24). Similar to that of PK, all modified sites were located in the conserved domain. Glucose-6-phosphate-dehydrogenase (G6PD) is a metabolic enzyme involved in the pentose phosphate pathway (supplemental Fig. S5); the enzyme exists in both *P. falciparum* and human hosts. Interestingly, no modification of *P. falciparum* G6PD was observed, but phosphorylation and 2-hydroxyisobutyrylation of G6PD of the human RBCs were identified on S84 and K95 (supplemental Dataset S24), respectively. The abundance of G6PD-K95^hib^ reached the highest point at 40 h (supplemental Dataset S24).

## Discussion

The results of this study and those reported earlier suggest that PTMs, widely present in all developmental stages of proteins of both *P. falciparum* and iRBCs, play an important role in the regulation of *Plasmodium* development. It is known that gene expression of *P. falciparum* during the IDC is highly periodic, with the majority of genes expressed in a “just-in-time” fashion, and the regulatory mechanisms are complex and remain largely uncharacterized (40). Here, we found that in the process of gene expression, the structural changes of nucleosomes containing histones were most closely related to acetylation. The transcription process and the initiation of translation were mainly regulated by phosphorylation. Translation elongation and ribosomal processing were jointly regulated by various types of modification.

Proteins with the same amino acid sequence may function in different cell compartments and at different time points. This study suggested that most PTMs in both *P. falciparum* and the iRBCs were tightly coordinated with the development of the parasite (Figs. 2 and 3). Of the six PTM types analyzed, phosphorylation and ubiquitination of parasite proteins displayed significant changes between *P. falciparum*-infected RBCs and the healthy RBCs, indicating that the two modifications have important roles in erythrocyte nesting p.i. Proteins with acetylation were predominantly located in the nucleus and ribosomes in *P. falciparum* and are likely involved in gene regulation processes, especially chromatin structural modulation and protein processing (Figs. 4 and 5). *N*-glycosylation mainly occurred on erythrocytic proteins involved in complement activation, the coagulation cascade, the fibrinolysis system, immune regulation, receptor binding, defense response, and lipid metabolism (Fig. 3). Protein *N*-glycosylation of the infected erythrocytes changed dramatically (Cluster 1 and Cluster 5 in Fig. 3) during the invasion stage of *P. falciparum* (48 h), such as *N*-glycosylation of MSP1, CR1, and basigin (supplemental Dataset S27).

The interaction between the parasite-derived ligands and host cell receptors has long been the focus of many studies, especially in the area of molecular pathogenesis. However, the influence of PTM on proteins in the interaction with host cell proteins has not been as thoroughly researched. Here, we systematically characterized PTMs of the pathogenesis-related proteins, including the parasite virulence factors. Malaria parasites invade RBCs under a cascade of receptor recognition, adhesion, and penetration processes that depend on the engagement of merozoite-derived ligands with the receptors on the surface of the human erythrocyte (6, 97). We found that PTMs might be closely related to this process, because PTMs of proteins derived from *P. falciparum* were identified, for example, the phosphorylation, acetylation, crotonylation, 2-hydroxyisobutyrylation, and *N*-glycosylation of MSPs; phosphorylation of RON2, RON4, GAPs, and MyoA; and ubiquitination, phosphorylation, and crotonylation of actin. Meanwhile, PTMs of proteins derived from RBCs were identified, for example, *N*-glycosylation of CR1 and basigin; *N*-glycosylation and ubiquitination of GPA; and phosphorylation, crotonylation, and 2-hydroxyisobutylation of GPC. Among these examples, the MSP family members were heavily modified at the early developmental stages of the parasite (Fig. 6), and it is postulated that PTMs can be critical for correct processing, structure formation, and translocation of the proteins within the parasite and the iRBCs.

Furthermore, it was interesting to find that *P. falciparum* actively stabilized certain human RBC proteins for the benefit of its development, probably via PTMs. For example, spectrin can bind to MSP1 to affect the RBC membrane curling ability of *P. falciparum* (98); actin filaments mediate PfEMP1 trafficking from Maurer's clefts to the RBC membrane (99). SLC4A1 is the major intrinsic membrane protein of red blood cells (100) and functions as a host receptor binding to MSP1 during the invasion into erythrocytes (101), and DMTN can bind to 14-3-3 involved in a large number of cellular processes (102). Furthermore, studies have found that iron bound to transferrin is the source of ferric ions for malaria parasites within mature erythrocytes (103). Here, the extent of PTMs, especially ubiquitination of these RBC structural proteins, was much lower in iRBCs than in RBCs, and the protein quantities were also greater in iRBCs than in RBCs. Thus, it could be hypothesized that the parasite stabilizes essential proteins for the purpose of its own development through selective dephosphorylation and deubiquitination.

Erythrocyte remodeling permits protein trafficking, harvesting of nutrients, and immune evasion. During the process of erythrocyte remodeling, the key molecules derived from both parasites and their host cells were extensively modified after protein translation. For example, PfEMP1 has been recognized as one of the virulence factors associated with severe malaria pathogenesis, and the modifications occurred in both the extracellular and intracellular domains, suggesting the critical roles of PTMs in both structural stability and molecular interaction of the functionally important protein family. PHIST proteins, central to host cell remodeling and thus of obvious importance in pathology, seem to be understudied; however, they underwent extensive modifications (supplemental Fig. S4). In addition, PTMs are widely distributed among the metabolically important enzymes involved in glycolysis and the pentose phosphate and purine salvage pathways. However, unlike in the homologous enzymes of *Trypanosoma* parasites, the modified amino acids were not located in the substrate-binding sites but rather were situated in the conserved domains of the enzymes, suggesting that PTMs might be involved in structural maintenance instead of enzymatic activity.

In conclusion, the establishment of the atlas of PTM-omics of *P. falciparum* and its host cells will promote a deeper understanding of parasite biology and parasite–host interaction, and the data also provide a candidate list in the search for antimalarials.

## Data availability

All data are available in the main text or in the supplementary materials. The MS spectrometry measurement files have been deposited at the ProteomeXchange consortium (http://proteomecentral.proteomexchange.org) via PRIDE Archive with the dataset identifier PXD017503. The annotated spectra have been uploaded as. apl files (zipped together) and msms.txt files through deposition of spectra to MS-Viewer (http://msviewer.ucsf.edu/prospector/cgi-bin/msform.cgi?form=msviewer) from the MaxQuant analysis output. The search key for the saved data set of phosphorylation (7680TPST) is jjkpcseur8, bpe3daeuba, and *dinjssnsco*; that of acetylation (7680TPAc) is Jloheksqtm, cwdf6yrf5b, and *bfecwmrask*; that of crotonylation (7680TPCrR2) is *kczfe72yfb*, *s43r9u2qmw*, and *kalnlkz4f4*; that of 2-hydroxyisobutyrylation (7680TP2o) is *4eeqwlcn4m*, *vjlbalu13q*, and *jb0c8p4yat*; that of ubiquitination (7680TPUb) is *uxqii9vo23*, *ugzxx9zcmn*, and *c8vh5zp9ep*; and that of *N*-glycosylation (7680TPNg) is *gin75pxhe3*. Additional data related to this article may be requested from the authors.

## Conflict of interest

The authors declare no competing interests.
