# Supplementary material for: Protein Modification Characteristics of the Malaria Parasite Plasmodium falciparum and the Infected Erythrocytes
Source: Mol Cell Proteomics. 2020 Nov 24;20:100001. doi: 10.1074/mcp.RA120.002375 (PMC7857547; doi:10.1074/mcp.RA120.002375)
Supplement: Supplemental Figures [file mmc32.pdf]

Supplemental Figures

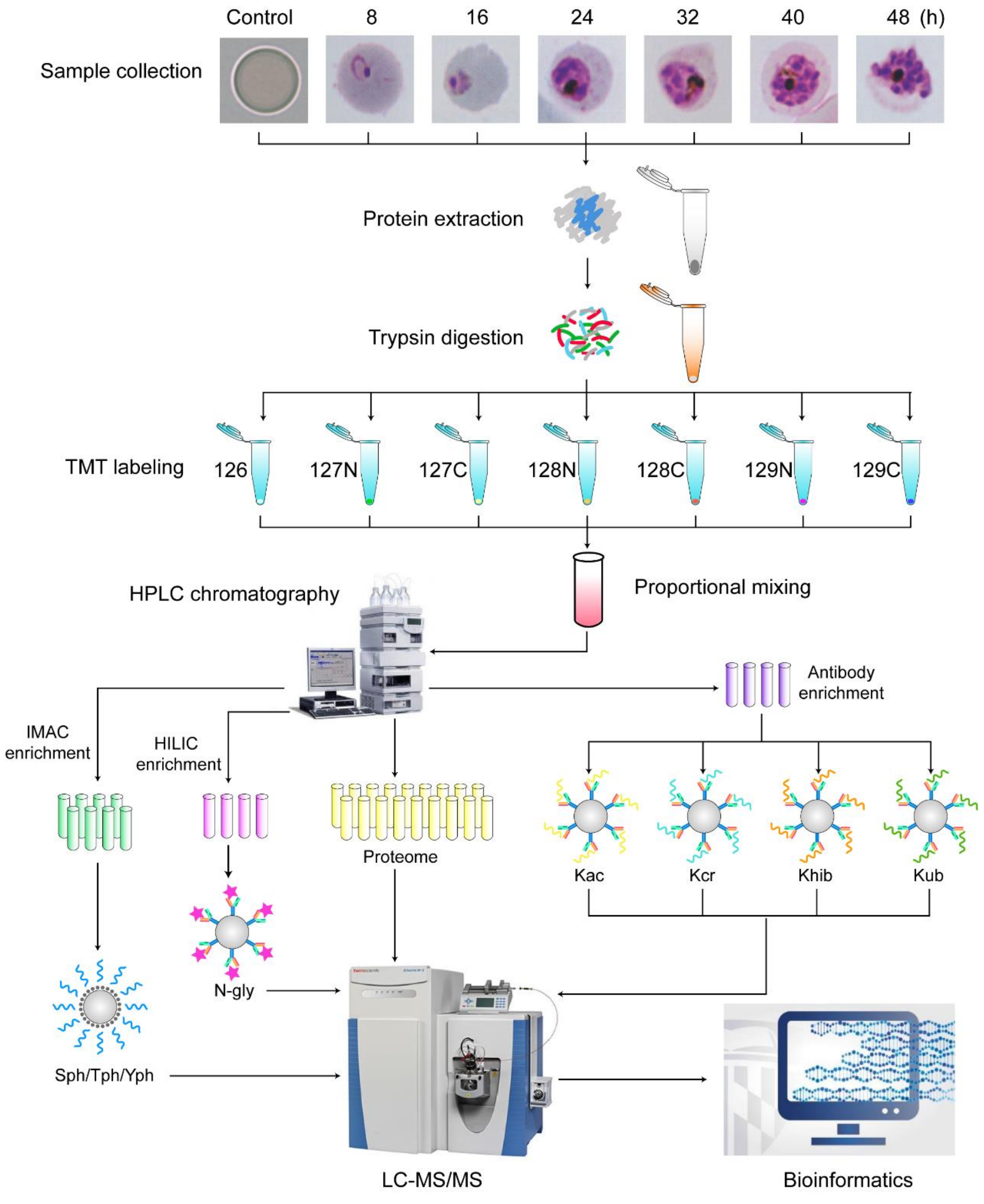

Fig S1. The workflow of TMT 10-plex global proteomic and six PTMomic analyses.

Plasmodium falciparum-infected red blood cells (pRBCs) were purified from a highly synchronized culture using the percoll-sorbitol purification method every 8 h during the IDC, taking normal RBCs as a control. The ProteoMiner^{TM} Protein Enrichment system was used for samples of the proteome to remove high-abundance proteins such as hemoglobin, but not for samples for PTMomes. Samples with three repetitions were treated by protein extraction, SDS-PAGE, western blotting analysis, pancreatin hydrolysis, tandem mass tag (TMT) labeling, high-performance liquid chromatography (HPLC), and affinity enrichment. Antibody-based affinity enrichment for acylation (acetylation, crotonylation, and 2-hydroxyisobutyrylation) and ubiquitination, and immobilized metal ion affinity chromatographic (IMAC) enrichment for phosphorylation and hydrophilic interaction chromatography (HILIC)-based enrichment for N-glycosylation were used. The PTM peptides were collected for LC-MS/MS analysis. The resulting MS/MS data were processed using the MaxQuant search engine (v.1.5.2.8). Tandem mass spectra were searched against SwissProt *Homo sapiens* and UniProt *Plasmodium falciparum* (isolate 3D7), along with reverse decoys and standard contaminant databases from MaxQuant. This search included quantitative research of seven omics, i.e., proteome, phosphorylation (Sph/Tph/Yph), acetylation (Kac), crotonylation (Kcr), 2-hydroxyisobutyrylation (Hib), N-glycosylation (Ng), and ubiquitination (Kub). Visualized figures were obtained via bioinformatics analysis.

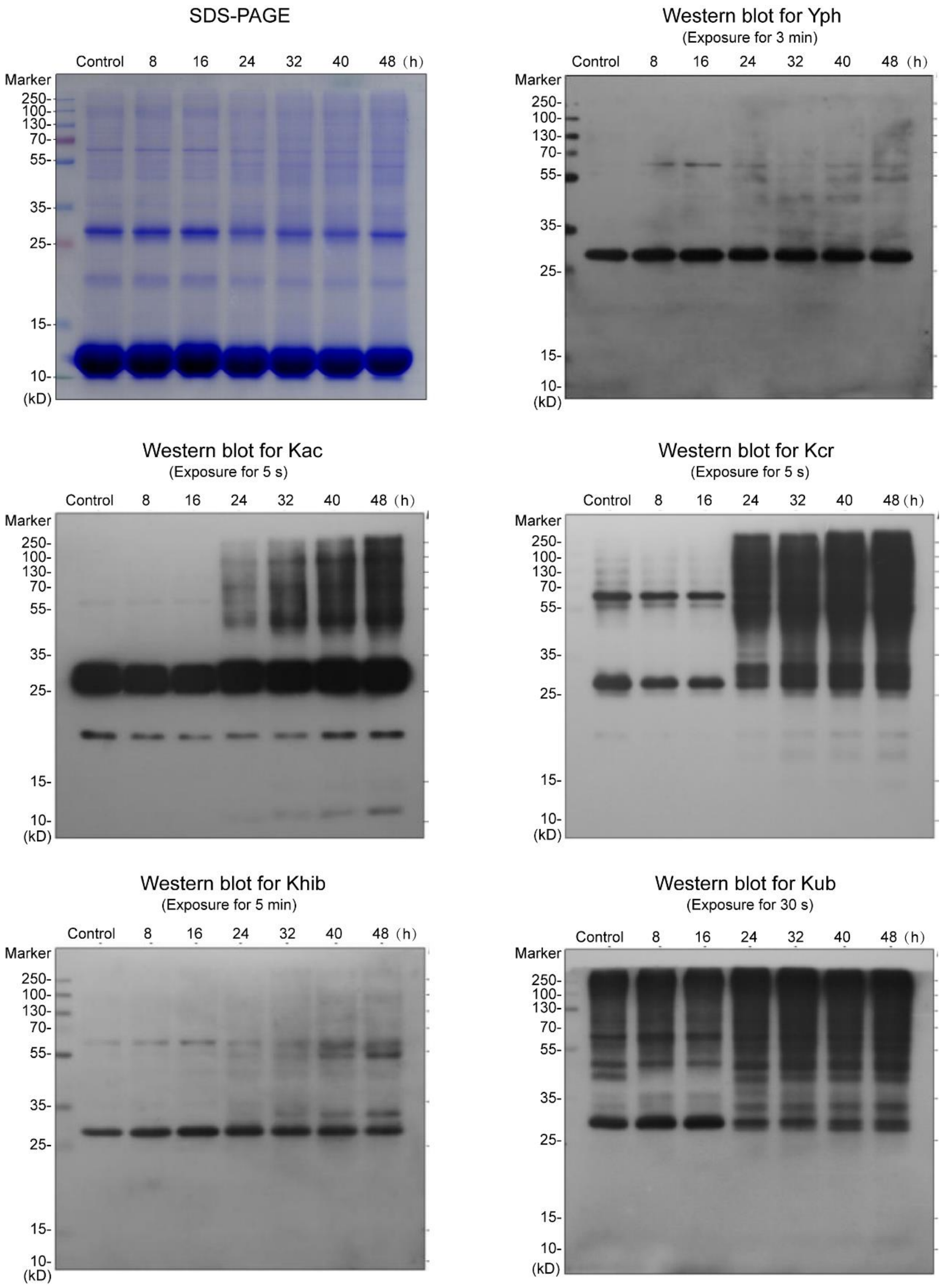

Fig S2. SDS-PAGE of protein samples for quality control and PTM antibody-based Western blotting analysis of whole-cell extracts.

SDS-PAGE (Sodium dodecyl sulfate-polyacrylamide gel electrophoresis) was used to detect the quality of protein samples. Antibodies included Goat anti-Mouse IgG (H+L) Secondary Antibody (HRP) and the pan anti-PTM antibodies for tyrosine phosphorylation (Yph), lysine acetylation (Kac), lysine crotonylation (Kcr), lysine 2-hydroxyisobutyrylation (Khib), and lysine ubiquitination (Kub). There were no antibodies for detecting N-glycosylation (Ng), threonine phosphorylation (Tph), or serine phosphorylation (Sph). The exposure times were 3 min for Yph, 5 s for Kac, 5 s for Kcr, 5 min for Khib, and 30 s for Kub.

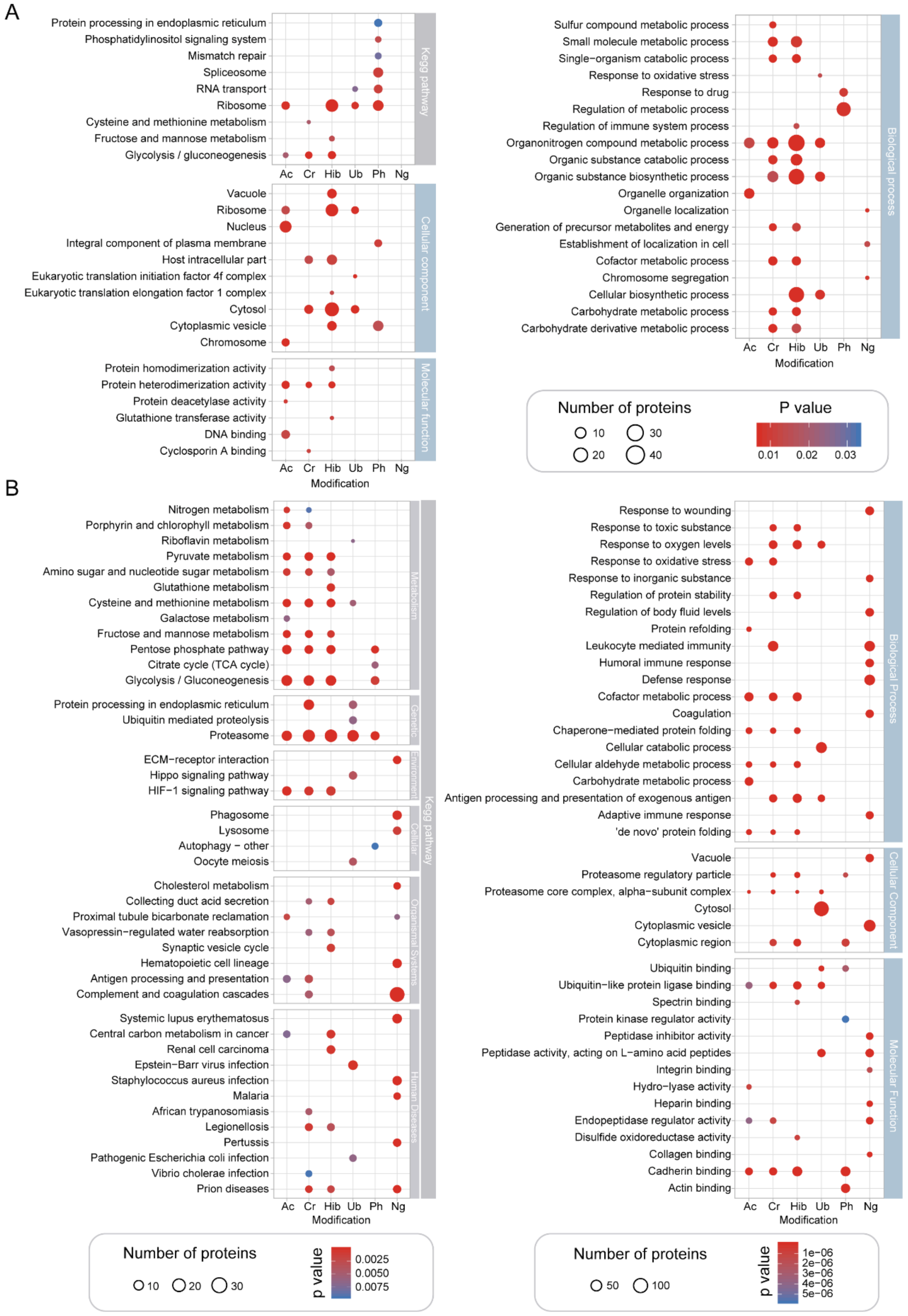

Fig S3. Functional enrichment of proteins modified.

(A) Functional enrichment of proteins for each type of modification in *P. falciparum*. The abscissa indicates the type of modification (phosphorylation (Ph), acetylation (Ac), crotonylation (Cr), 2-

hydroxyisobutyrylation (Hib), N-glycosylation (Ng), and ubiquitination (Ub). The ordinate indicates function entries enriched by KEGG pathways ($P < 0.05$) and GO terms (including cellular component, molecular function, and biological process; $P < 0.05$). The number of proteins enriched in each modification is indicated by the size of the bubble. The significance degree of the enrichment is represented by the *P*-value. The smaller the *P*-value, the redder the bubble, and the more significant the functional enrichment. The larger the *P*-value, the bluer the bubble, and the less significant the functional enrichment. (B) Functional enrichment of proteins modified by each modification in RBCs. The abscissa indicates the type of modification (Ph, Ac, Cr, Hib, Ng, and Ub). The ordinate indicates function entries enriched by KEGG pathways (including Metabolism, Genetics, Environment, Cellular, Organismal Systems, Human Diseases; $P < 0.01$) and GO terms (including cellular component, molecular function, biological process, $P < 0.00001$). The number of proteins enriched in each modification is indicated by the size of the bubble. The significance degree of enrichment is represented by the *P*-value. The smaller the *P*-value, the redder the bubble, and the more significant the functional enrichment. The larger the *P*-value, the bluer the bubble, and the less significant the functional enrichment.

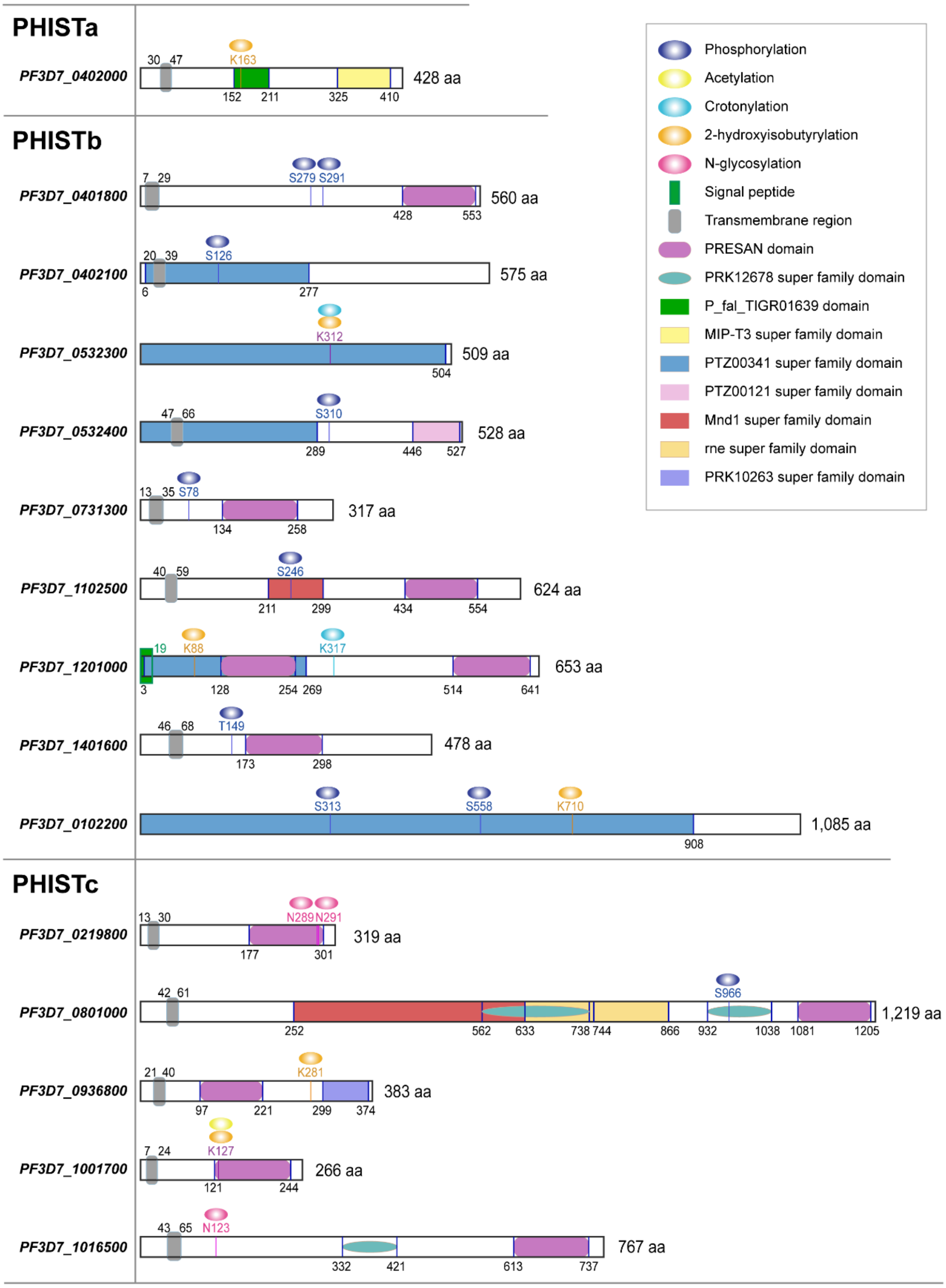

Fig S4. The posttranslational modifications of PHIST proteins in *P. falciparum*.

PHIST proteins comprise three distinct subgroups (termed PHISTa, PHISTb, and PHISTc) that are distinguished by the presence and position of several conserved tryptophan residues. The

conserved domains and modification sites of PHIST proteins are shown in the figure. The conserved domains are the PRESAN domain, the PTZ00341 superfamily domain, the P_fal_TIGR01639 domain, the MIP-T3 superfamily domain, the PTZ00121 superfamily domain, the Mnd1 superfamily domain, the PRK12678 superfamily domain, the rne superfamily domain, and the PRK10263 superfamily domain.

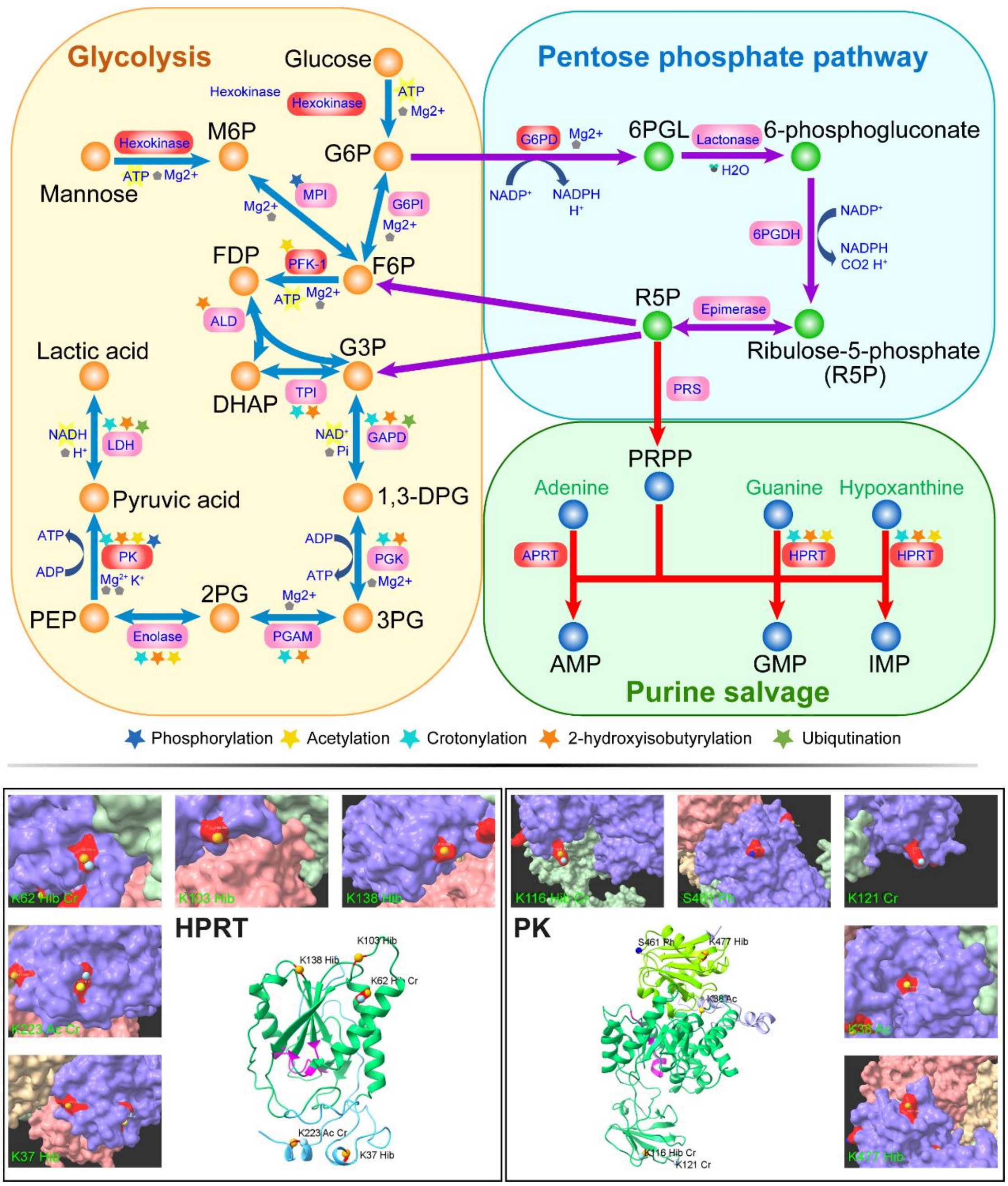

Fig S5. Metabolic pathways and key enzymes regulated by PTMs.

The yellow plate illustrates the glycolysis pathway. The blue plate shows the pentose phosphate pathway. The green plate shows the purine salvage synthesis pathway. The proteins in pink shades indicate catalytic enzymes, and the red shades indicate that the corresponding protein is the rate-limiting enzyme. The pentagrams refer to modification types. The image section below shows the 3D structures of two key

enzymes, and the modification sites are marked. The 3D structure was prepared using Pymol. The surface structure of each modification site, which was prepared using ChimeraX, is displayed on the periphery. G6P: glucose 6-phosphate; M6P: mannose 6-phosphate; F6P: fructose 6-phosphate; FDP: fructose-1, 6-diphosphate; DHAP: dihydroxyacetone phosphate; G3P: glyceraldehyde 3-phosphate; 1,3-DPG: 1,3-Disphosphoglycerate; 3PG: 3-phosphoglycerate; 2PG: 2-phosphoglycerate; PEP: phosphoenolpyruvate; G6PI: glucose-6-phosphateisomerase; MPI: phosphomannose isomerase; PFK-1: phosphofructokinase; ALD: aldolase; TPI: triose-phosphate isomerase; GAPD: glyceraldehyde-3-phosphate dehydrogenase; PGK: phosphoglycerate kinase; PGAM: phosphoglycerate mutase; PK: pyruvate kinase; LDH: lactate dehydrogenase; 6PGL: *6*-phosphogluconolactone; R5P: Ribulose 5-phosphate; G6PD: Glucose-6-phosphate-dehydrogenase; 6PGDH: 6-phosphaogluconate dehydrogenase; PRS: phosphoribosylpyrophosphate synthetase; PRPP: phosphoribosylpyrophosphate; AMP: Adenosine monophosphate; GMP: guanosine monophosphate; IMP: inosine monophosphate; APRT: Adenine phosphoribosyltransferase; HPRT: hypoxanthine-guanine phosphoribosyltransferase.
